# Intrastate inequality of COVID-19 vaccination coverage: spatial analysis and socioeconomic, Santa Catarina, 2021-2023

**DOI:** 10.1590/S2237-96222025v34e20240329.en

**Published:** 2025-08-08

**Authors:** Clorine Borba Zanlourensi, Antônio Fernando Boing

**Affiliations:** 1 Universidade Federal de Santa Catarina, Centro de Ciências da Saúde, Programa de Pós-Graduação em Saúde Coletiva, Florianópolis, SC, Brazil

**Keywords:** Vaccination, COVID-19, Health Inequality Indicators, Sociodemographic Factors, Public Health, Vacunación, COVID-19, Indicadores de Desigualdad en Salud, Factores Sociodemográficos, Salud Pública

## Abstract

**Objective:**

To analyze the distribution of COVID-19 vaccination coverage in Santa Catarina, Brazil, and its association with socioeconomic aspects of the state’s municipalities between 2021 and 2023.

**Methods:**

We analyzed data on individuals aged 12 and over, with a full three-dose vaccination schedule. The independent variables included educational data, municipal human development and racial composition of municipalities. We analyzed the evolution and spatial distribution of vaccination coverage, identifying clusters of municipalities and calculating absolute ratios and differences between the highest and lowest quintiles of vaccination coverage.

**Results:**

Vaccination coverage in the state as a whole was 26.9% for adolescents, 50.3% for adults and 78.8% for the elderly. Municipalities with higher vaccination coverage had 5.4 times more people vaccinated in the 12-17 age group, 2.1 times more in the 18-59 age group and 1.4 times more in the population aged 60 and over. Municipalities with a higher educational level, a lower Black population proportion and a lower municipal human development index had higher vaccination coverage. These disparities were persistent. Clusters of municipalities with high vaccination were found.

**Conclusion:**

There were disparities in the state’s vaccination coverage, with association between socioeconomic aspects of the municipalities.

## Resumen

**Objetivo:**

Analizar la distribución de la cobertura de vacunación contra Covid-19 en Santa Catarina, Brasil, y su asociación con aspectos socioeconómicos de los municipios entre 2021 y 2023.

**Métodos:**

Se analizaron datos de personas de 12 años y más, con esquema completo de vacunación con tres dosis. Las variables independientes incluyeron datos educativos, desarrollo humano municipal y composición racial de los municipios. Se analizó la evolución y distribución espacial de la cobertura de vacunación, identificando grupos (clusters) de municipios y calculando razones absolutos y diferencias entre los quintiles más altos y más bajos de cobertura de vacunación.

**Resultados:**

La cobertura de vacunación en el estado fue de 26,9% para jóvenes, 50,3% para adultos y 78,8% para adultos mayores. Los municipios con mayor cobertura de vacunación tuvieron 5,4 veces más población vacunada entre 12 y 17 años, 2,1 veces más en el grupo de 18 a 59 años y 1,4 veces más en la población de 60 y más años. Los municipios con mayor nivel educativo, menor proporción de población negra y menor índice de desarrollo humano municipal tuvieron mayor cobertura de vacunación. Estas disparidades eran persistentes. Se encontraron clusters de municipios con altas tasas de vacunación.

**Conclusión:**

Existieron disparidades en la cobertura de vacunación en el estado, con asociación entre aspectos socioeconómicos de los municipios.

**Ethical aspects**

This research used public domain anonymized databases.:

## Introduction

Vaccination against COVID-19 is considered a crucial public health measure to reduce hospitalization and mortality rates from this disease (1). Vaccines have shown high efficacy in preventing severe forms of COVID-19. This shows that large-scale vaccination played a fundamental role in controlling the COVID-19 pandemic and in individual protection (2). In addition to the primary vaccination schedule, with two doses, booster doses were recommended to prevent severe and fatal COVID-19 in 2021 for all age groups (3). 

By May 2024, it was estimated that 67.0% of the world’s population had completed the primary series and 32.0% had received at least one booster dose of COVID-19 vaccine (4). In addition to being low, this vaccination coverage was considered unequal, with the percentage of two-dose coverage reaching 79.86% among people living in high-income countries and 32.82% of the population in low-income countries (5).

In Brazil, from 2020 to 2022, there was a marked national lack of coordination in actions to combat COVID-19, and many procedures were not guided by the best scientific evidence. Even though Brazil had a National Immunization Program, internationally recognized as one of the most complete and widespread in the world, there were delays in acquiring COVID-19 vaccines and a strong campaign to discredit vaccination (6). By May 2024, 84.6% of Brazilians had completed the primary vaccination schedule, while 52.8% had received a booster dose, with strong variation according to the age group of the population (7). Despite the Brazilian National Health System principle of equity, access to health services in Brazil presented regional, socioeconomic and demographic inequalities (8).

The scarce literature that has investigated inequalities in COVID-19 vaccination in Brazil has been concentrated on analyses of the entire national territory, with few studies with intra-urban and intra-state spatial analyses (8-10). Indicators that estimate averages for larger geographic clusters can hide important disparities within them. Analyses using data that is more aggregated and divided only into large macro-regions can hide significant inequalities, such as between different municipalities or regions within states.

Gaining knowledge about regional disparities and understanding them is fundamental to designing more effective public policies and promoting a more equitable supply of inputs and public health actions. In June 2024, Santa Catarina ranked 17^th^ in vaccination coverage among the 27 Brazilian federative units. No studies were found that systematically evaluated its intrastate distribution (11). This gap makes it impossible to analyze the government’s role in complying with the principle of equity in the health system, especially in times of health emergencies.

The objective of this study was to analyze the distribution of COVID-19 vaccination coverage in Santa Catarina and its association with socioeconomic aspects of the state’s municipalities between 2021 and 2023.

## Methods

### Design and setting

This was an observational, ecological and longitudinal study. The data obtained refers to vaccination against COVID-19 in the municipalities of Santa Catarina between January 16, 2021 and June 3, 2023.

### Participantes

All individuals aged 12 and over living in Santa Catarina and vaccinated between January 16, 2021 and June 3, 2023 were included in the study. The records were classified into three age groups: adolescents (12-17 years old), adults (18-59 years old) and elderly people (60 years old or over).

### Variáveis

The dependent variable was full vaccination with three doses: first dose, second dose and booster dose. The records were classified according to epidemiological week, the order in which vaccines were administered and municipality of residence.

The independent variables were sociodemographic indicators. The education indicator used was the number of years of study expected up to 18 years of age among residents of each municipality, under the premise that the schooling patterns observed when data were collected would be maintained throughout their lives. The racial composition of each municipality was determined by the proportion of Black residents in relation to the total number of residents. The Municipal Human Development Index (MHDI) is a measure that evaluates human development in each municipality, considering three main indicators: education, longevity and income. These variables were divided into quintiles based on their distribution in the municipalities.

### Data sources

We accessed vaccination records obtained from the National Immunization Program Information System (*Sistema de Informações do Programa Nacional de Imunizações* - SI-PNI). In Brazil, all doses of vaccines administered against COVID-19 are compulsorily recorded by health services for transmission to the Ministry of Health. This consolidates data from the entire national territory and makes it publicly available on the openDataSUS platform. All databases contain anonymized information regarding administration of vaccine doses. This data set includes information about the municipality and state, date of vaccine administration, type of vaccine administered, sex and age of each person vaccinated. Vaccination data can be accessed in the following repository: https://github.com/covid19br/dados-vacinas. 

In order to calculate vaccination coverage, the dataset on the resident population was used as the denominator. This dataset was provided by the Brazilian Institute of Geography and Statistics, and referred to the 2010 Demographic Census (https://sidra.ibge.gov.br/tabela/1378) e 2022 (https://sidra.ibge.gov.br/tabela/9514).

The sociodemographic indicators were obtained from the 2010 Demographic Census, that being the most recent year with consolidated municipal socioeconomic data available in Brazil in June 2024. These indicators were published in the Brazilian Human Development Atlas (https://www.atlasbrasil.org.br/). 

### Bias

By using secondary data, this study may have inconsistencies and errors arising from failures in vaccination information recording on the SI-PNI. Having used the 2010 Demographic Census, the most recent available at the time of data collection, may have resulted in inaccuracies due to demographic and other changes since 2010.

### Study size

The study included all COVID-19 vaccination records for individuals over 12 years of age, which were available in the SI-PNI, for the period from January 16, 2021 to June 3, 2023. Data on individuals aged 0-11 years were excluded from the database

### Statistical methods

In order to calculate vaccination coverage, the number of vaccinated people was divided by the resident population in each age group within each municipality, and the result was multiplied by 100. The three complete dose values were estimated for all epidemiological weeks from 2021 until the 22^nd^ epidemiological week of 2023 (January 16, 2021 to June 3, 2023).

Maps of the spatial distribution of vaccination coverage, according to the municipalities of Santa Catarina, were produced using Quantum Geographic Information System software (QGIS), version 3.34.0. 

The municipalities were divided into quintiles according to sociodemographic variables, and vaccination coverage was calculated in each age group. Each quintile represents 20.0% of the distribution of vaccination coverage, from the lowest to highest. The ratio of the outcome according to the quintiles was also calculated – by dividing the vaccination coverage value of the highest quintile by the value of the lowest quintile – and the absolute difference – by subtracting the vaccination coverage of the quintile with the highest value from that observed in the quintile with the lowest value.

In order to investigate the existence of spatial clusters, we calculated the univariate local Moran’s index (Local Indicators of Spatial Association – LISA). This method analyzed data spatial autocorrelation, identifying regions in which similar values ​​are closer to each other than would be expected by chance.

Four statistically significant classes of clusters were identified: i) high-high, in which there is intensity of vaccination coverage between an area and its neighboring areas; ii) low-low, in which there is low vaccination coverage between an area and its neighboring areas; iii) low-high, which reflects areas with low vaccination coverage values ​​surrounded by areas with high values; and iv) high-low, which indicates an isolated point of high vaccination coverage, in contrast to the surrounding pattern. We used GeoDa 1.22.0.4 to perform spatial analysis and create maps. The statistical analyses were performed using Stata 15.1.

## Results

As at epidemiological week 22, 2023, 14,872,425 doses of COVID-19 vaccine had been administered to residents of Santa Catarina. The elderly were the group with the highest three-dose vaccination coverage (VC) (78.8%, VC 10.7%), followed by adults (50.3%, VC 15.1%) and adolescents (26.9%, VC 18.6%).

**Figure 1:**
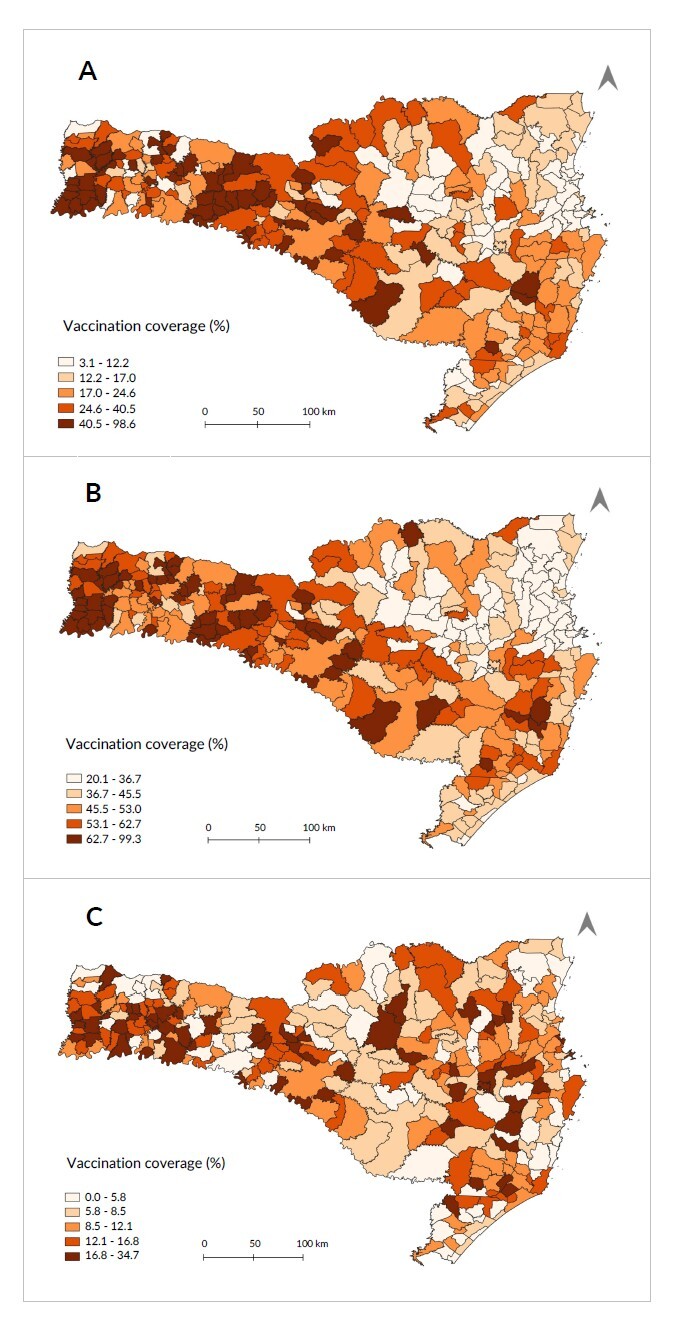
COVID-19 vaccination coverage, by age categories. Santa Catarina, Epidemiological Week 22, 2023a (n=295)

Three-dose vaccination coverage varied substantially between municipalities. The West Santa Catarina macro-region stood out, especially the micro-region of São Miguel do Oeste, with the highest coverage rates in municipalities in the three age categories. The North Santa Catarina and Vale do Itajaí macro-regions recorded the lowest coverage rates (Figure 1). The geographic divisions of each macro-region of the state are shown below (Supplementary Figure 1) and vaccination coverage is presented throughout the period of analysis, noting that regional differences appeared from the beginning of the vaccination campaign and continued until the end of the period analyzed (Supplementary Figure 2).

(A) population aged 12-17 years; (B) population aged 18-59 years; (C) population aged 60 years or over; aEpidemiological week 22, 2023, refers to June 3, 2023. Santa Catarina, 2023, 295 municipalities.

**Figure 2:**
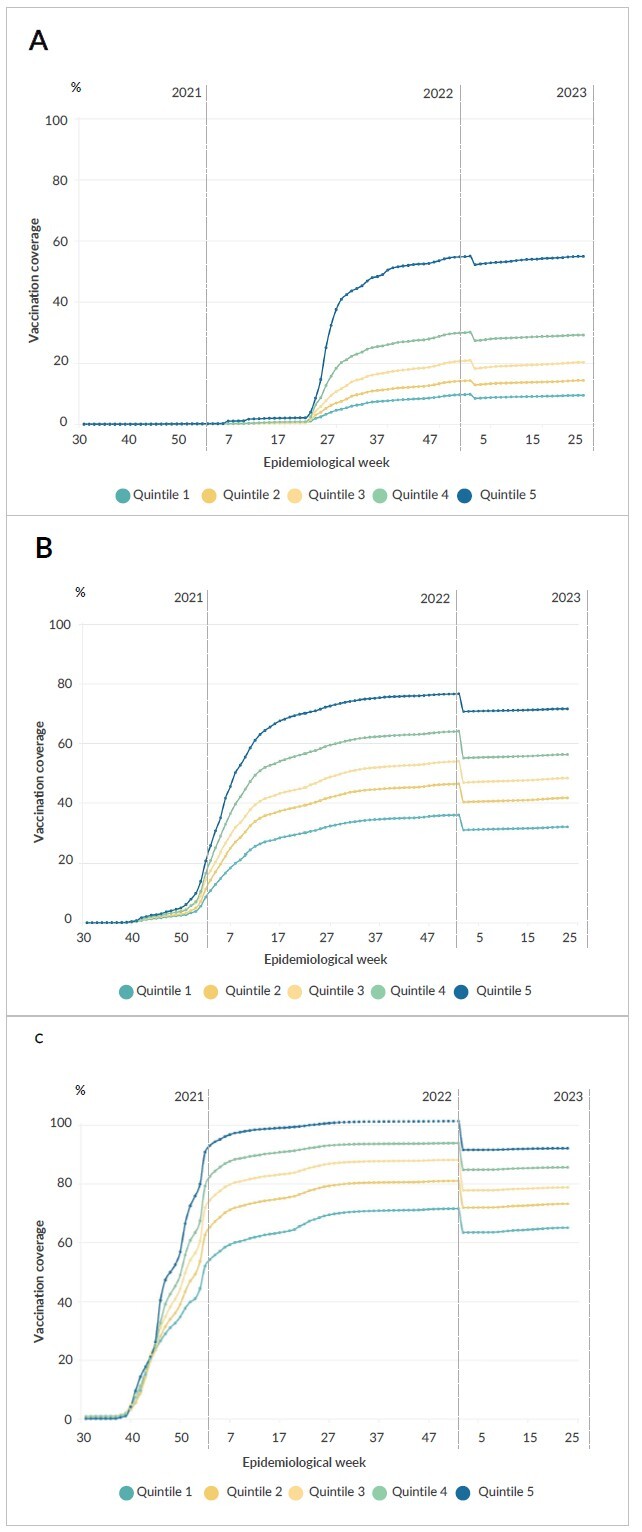
Evolution of COVID-19 vaccination coverage, by municipality vaccination coverage^a^. Santa Catarina, 2021-2023 (n=295)

(A) population aged 12-17 years; (B) population aged 18-59 years; (C) population aged 60 years or over; aQuintile: data divided into five parts that represent 20% of distribution, from the lowest to the highest (from the 1st to the 5th quintile). Santa Catarina, 2023, 295 municipalities.

There was profound disparity in vaccination coverage between municipalities when grouped into coverage quintiles (Table 1). Quintile 1, being that with the lowest vaccination coverage, accounted for 10.8% of 12-17 age group with three doses, 36.2% for the 18-59 age group and 73.1% for individuals aged 60 or over. Quintile 5 comprised the municipalities with the highest vaccination coverage. This quintile had values ​​equal to 58.2% among those aged 12-17, 77.5% for those aged 18-59 and 104.2% for those aged 60 or over. The municipalities in the quintile with the highest expected schooling at age 18 had the highest vaccination coverage in all age groups. Greater vaccination coverage was also observed in quintiles of municipalities with a lower percentage of Black people. The pattern was different with regard to the MHDI; quintile 5, i.e. the quintile with the highest MHDI, had the highest vaccination coverage.

**Table 1:** Proportion of COVID-19 vaccination coverage by municipalities in quintile groups, and socioeconomic indicators. Santa Catarina, Epidemiological Week 22, 2023^a^ (n=295)

Socioeconomic variable	12-17 years n (%)	18-59 years n (%)	60+ years n (%)
**Municipalities**			
Quintile^b^ 1 (lowest)	11,132 (10.8)	612,836 (36.2)	272,298 (73.1)
Quintile^b^ 2	29,811 (16.0)	429,590 (46.1)	262,103 (81.5)
Quintile^b^ 3	31,643 (23.2)	585,289 (54.9)	211,169 (88.2)
Quintile^b^ 4	15,819 (32.9)	262,442 (63.5)	88,222 (94.6)
Quintile^b^ 5 (highest)	10,928 (58.2)	113,840 (77.5)	50,108 (104.2)
**Years of schooling expected at 18 years old**			
Quintile^b^ 1 (lowest)	11,366 (20.8)	191,315 (44.8)	87,175 (82.0)
Quintile^b^ 2	26,943 (17.5)	553,324 (42.9)	244,354 (79.1)
Quintile^b^ 3	27,930 (18.5)	610,320 (47.6)	270,421 (83.2)
Quintile^b^ 4	23,868 (24.6)	500,708 (57.2)	206,580 (85.0)
Quintile^b^ 5 (highest)	8,890 (29.5)	142,183 (58.4)	71,635 (91.2)
**Proportion of Black people**			
Quintile^b^ 1 (lowest)	6,868 (28.8)	102,505 (54.2)	54,668 (86.1)
Quintile^b^ 2	9,741 (22.2)	173,850 (51.1)	84,807 (84.4)
Quintile^b^ 3	14,595 (22.2)	272,878 (51.1)	125,656 (84.7)
Quintile^b^ 4	20,376 (19.8)	401,075 (46.7)	183,416 (83.6)
Quintile^b^ 5 (highest)	47,753 (18.9)	1,053,689 (47.8)	435,353 (81.4)
**Municipal Human Development Index** (MHDI)			
Quintile^b^ 1 (lowest)	12,199 (25.5)	208,761 (53.7)	87,150 (84.4)
Quintile^b^ 2	10,737 (20.3)	200,921 (48.3)	97,046 (83.4)
Quintile^b^ 3	12,475 (19.4)	240,075 (46.6)	110,466 (81.6)
Quintile^b^ 4	16,212 (21.7)	302,754 (49.7)	137,219 (85.5)
Quintile^b^ 5 (highest)	47,294 (19.2)	1,043,603 (47.8)	447,164 (82.0)

^a^Epidemiological week 22, 2023, refers to June 3, 2023; ^b^Quintile: data divided into five parts that represent 20% of vaccination coverage distribution, from the lowest to the highest (from the 1^st^ to the 5^th^ quintile). Santa Catarina, 2023, 295 municipalities.

Absolute inequalities and the ratio between the lowest and highest quintiles of sociodemographic indicators, throughout the vaccination campaign, revealed significant disparities, especially between the lowest and the highest quintiles (Table 2). Municipalities with the highest vaccination coverage demonstrated that they had 5.4 times more people vaccinated among those aged between 12 and 17 years, 2.1 times more in the 18-59 age group and 1.4 times more in the population aged 60 and over. The years of study socioeconomic indicator showed greater inequality in vaccination coverage across all age categories, especially among young people aged 12-17 (Table 2).

**Table 2:** Ratio, relative and absolute differences of COVID-19 vaccination coverage by age strata, comparing the values of the highest and lowest quintiles (quintile 5 and quintile 1) for vaccination coverage in the municipalities and socioeconomic indicators. Santa Catarina, Epidemiological Week 22, 2023^a^ (n=295)

Socioeconomic indicators (years)	Vaccination coverage ratio (Q5^b^/Q1^c^)	Absolute difference of vaccination coverage (%) ( Q5^b^/Q1^c^)
Municipalities		
12-17	5.39	47.4
18-59	2.14	41.3
60+	1.43	31.1
**Expected years of schooling at 18 years old**		
12-17	1.42	8.7
18-59	1.30	13.6
60+	1.11	9.3
**Proportion of Black people**		
12-17	0.66	-9.9
18-59	0.88	-6.5
60+	0.95	-4.7
**Municipal Human Development Index** (MHDI)		
12-17	0.75	-6.4
18-59	0.89	-6.0
60+	0.97	-2.3

^a^Epidemiological week 22, 2023, refers to June 3, 2023; ^b^Q5: quintile 5; ^c^Q1: quintile 1. Santa Catarina, 2023, 295 municipalities.

During the vaccination campaign, the quintiles of municipalities that recorded the highest vaccination coverage at the beginning remained ahead with higher coverage than those with lower coverage at the beginning, regardless of age group (Figure 2). The same pattern was observed when coverage was analyzed according to sociodemographic variables (Supplementary Figure 3).

**Figure 3:**
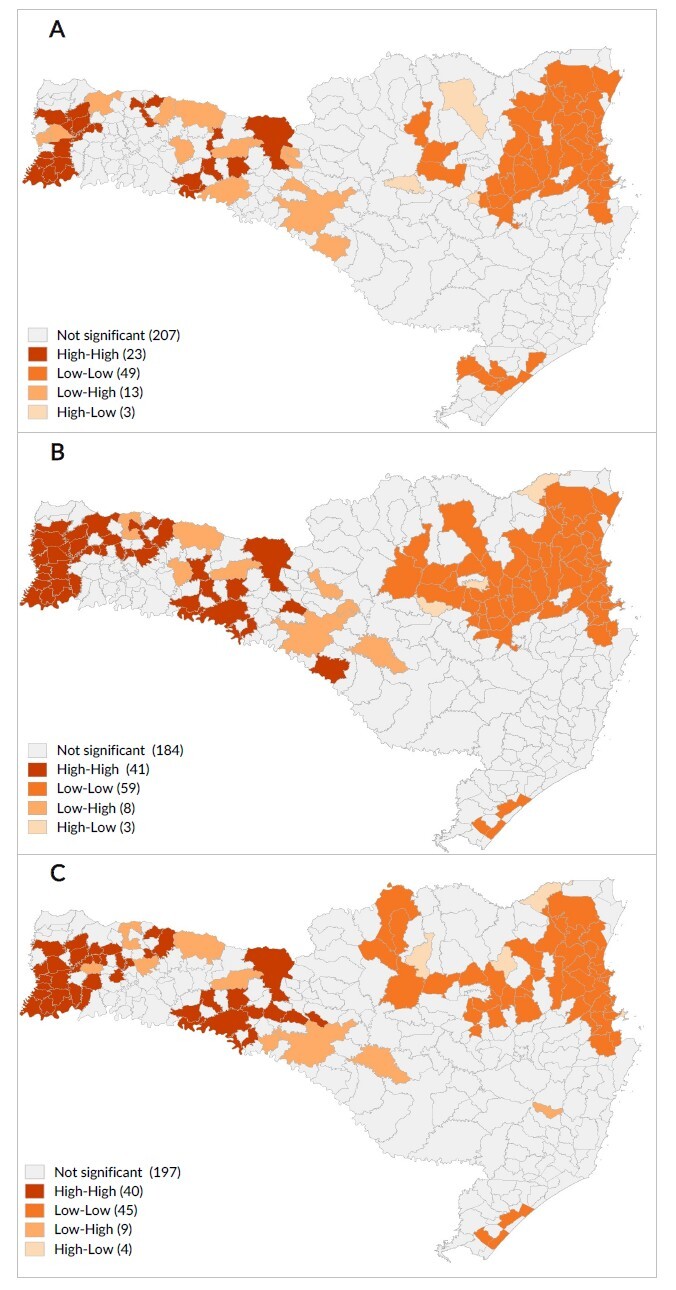
Spatial autocorrelation of COVID-19 vaccination coverage, according to univariate local Moran’s index. Santa Catarina, Epidemiological Week 22, 2023^a^ (n=295)

(A) population aged 12-17 years; (B) population aged 18-59 years; (C) population aged 60 years or over. aEpidemiological week 22, 2023, refers to June 3, 2023. Santa Catarina, 2023, 295 municipalities.

Spatial autocorrelation showed statistically significant high-high clusters, which indicated high vaccination coverage rates in areas where neighboring areas also had high values ​​(Figure 3). These clusters were mainly located in the West Santa Catarina macro-region, covering all age groups. The low-low clusters, which indicate low vaccination coverage with neighboring areas in similar conditions, were mainly concentrated in the North Santa Catarina and in the Itajaí Valley macro-regions.

## Discussion

This study identified significant disparities in COVID-19 vaccination coverage between municipalities of Santa Catarina. Municipalities with a higher educational level, lower proportion of Black people and lower MHDI had higher COVID-19 vaccination coverage. When comparing municipalities, vaccination inequality was higher among young people and lower among the elderly. These disparities remained persistent from the beginning to the end of the period investigated. Clusters of municipalities with high vaccination rates were found in the West Santa Catarina macro-region and with low vaccination rates in the North Santa Catarina and Vale do Itajaí macro-regions.

Vaccination coverage discrepancy also occurred between different regions and municipalities in Brazil as a whole (8,12). Brazilian municipalities presented the level of aggregation that concentrated most variability in this outcome in comparison to states and regions, mainly with regard to the third dose of the vaccine (8). In this study, inequalities between municipalities were consistent across all age groups assessed and persisted throughout all epidemiological weeks. Clusters of municipalities with higher and lower vaccination coverage were also found. This may indicate that some municipalities share similar spatial characteristics that favor high coverage, while others face common challenges that hinder vaccination.

These aspects may be related to health service infrastructure, which may have hampered the logistics of the vaccination campaign and access to services in some locations (13). Inadequate healthcare facilities may be associated with lack of sufficient financial resources to invest in better structures, equipment and staff training (14). Brazilian regions with poorer income indicators may encounter difficulties in guaranteeing adequate resources for health infrastructure, which may hinder effective and accessible vaccine administration for the population (12). 

There are other obstacles, such as access to health services and resistance to vaccination among the local population (13,15). Residents of Brazilian municipalities with lower incomes may have faced greater financial challenges when trying to access vaccination sites. There may also be lower levels of trust in health authorities and, consequently, in recommendations to get vaccinated (12). A survey carried out in 14 countries demonstrated that access to preventive health services is positively associated with a higher rate of vaccination against COVID-19 (16). Vaccination may be influenced by the quality of recent experiences of health care service users (16).

Health professionals play a crucial role in disseminating reliable information about vaccination (16,17). Health service users’ trust in the care they receive strengthens the bond with the healthcare team, encouraging them to seek healthcare services and participate in vaccination campaigns (13). Lack of sufficient human resources and training opportunities for health professionals can cause difficulties in administering doses, as well as in maintaining records on the SI-PNI (15).

Operationalization of vaccination campaigns in Brazil occurs at the municipal level, which, primarily, makes it the responsibility of municipal governments to identify and overcome obstacles in population immunization. Although this local responsibility is important, active participation of state governments plays a fundamental role in the success of campaigns. In addition to improving vaccine distribution and identifying municipalities with poor vaccination coverage, state governments also need to offer necessary technical and financial support (8). The gap that occurred in actions during the years of the health emergency needs to be highlighted in order to mitigate inequalities in vaccination coverage, which were persistent over time. To address these disparities, it is essential that state governments actively engage in the vaccination process, coordinating efforts and allocating resources efficiently to ensure comprehensive and equitable vaccination coverage across each state. This action is essential to identify regional clusters that present challenges and require a differentiated approach.

In the context of socioeconomic inequalities, a variety of countries showed association between higher COVID-19 vaccination rates and higher levels of education (18,19). In Brazil, up until 2022, population coverage of booster doses was higher in municipalities with a higher expected level of education at age 18 (58.7%) compared to those with a lower educational level (41.2%) (8). The findings of our study corroborate those of other studies presented so far, as, in all age groups, disparities were found between municipalities according to education level. The relationship between population education and vaccination coverage can be explained by the greater ability of people with a higher level of education to access health information and trust more in science and public health recommendations, which can increase their willingness to get vaccinated (20,21).

Municipalities with a higher proportion of people with higher levels of education can have higher vaccination coverage. Brazilian adolescents and adults with a lower educational level have greater vaccination hesitancy (22). Municipalities in which the population has higher levels of education may also be those with a better existing health service structure, fewer access barriers, better prepared health professionals, thus offering objective and material conditions that are more conducive to vaccination. One of the strategies for disseminating of scientifically based knowledge about COVID-19 vaccines to the entire population is posting this information in traditional and electronic media and producing support materials, such as videos and booklets (23).

Our study revealed that municipalities with a higher proportion of Black people had lower vaccination coverage. In Brazil as a whole, municipalities with a higher proportion of Black people were more likely to have lower vaccination coverage (8,12). In Michigan, in the United States, neighborhoods with a higher proportion of Black people also had lower COVID-19 vaccination rates (24). Places with a higher proportion of Black people may have higher levels of poverty, unemployment, low education and less access to health services, including availability of vaccination sites and transport. All of these factors can affect access to and demand for vaccination (25). The engagement of the three levels of Brazilian National Health System management in expanding vaccine rooms, active tracing and mobilization of health professionals in places with greater social inequality can be effective strategies for achieving equitable access to vaccination (23).

Vaccination coverage in municipalities with the highest MHDI was lower when compared to those with the highest MHDI. In the state of Paraíba, the highest percentage of hospitalized individuals with a full vaccination schedule occurred in municipalities with low and medium MHDI (26). An analysis of 5,570 Brazilian municipalities showed that vaccination coverage was lower in places with low MHDI. In municipalities with a solid primary health care network, vaccination coverage can come close to levels found in areas with high MHDI, highlighting the importance of primary care for improving vaccination in less developed regions (27).

MHDIs can hide disparities within regions, where some areas face high levels of inequality and unequal access to health services. Municipalities with lower MHDI, but with well-targeted vaccination campaigns, can achieve higher coverage rates (28). The MHDI is comprised of longevity, education and income indicators. However, it is possible for a municipality to have a high MHDI due to longevity and income, but face significant challenges in terms of education, and vice versa. This complexity may help explain the results of our study with regard to MHDI (28).

This study had some limitations. Data on racial and socioeconomic composition came from the 2010 Demographic Census, the most recent year available at the time of our analysis. Municipality indicators may have improved or worsened comparatively over the following decade and they may have been ranked in a quintile that is different from their current scenario. Vaccine dose recording by municipalities faced problems during the COVID-19 pandemic, such as delays and errors in filling out records due to lack of adequate health professional training and monitoring on the importance and operationalization of data, problems with local information systems and data centralization, as well as limited internet access in remote areas. We used Brazil’s official vaccination data and, although there is a need for improvement, SI-PNI data is the most complete and reliable data available, and is continuously monitored to identify and correct errors. The information is made available in a transparent manner and enables comprehensive monitoring of vaccination campaigns.

Analysis of disparities in COVID-19 vaccination coverage in Santa Catarina revealed worrying patterns. These disparities have persisted over time, indicating that they are structural issues that require ongoing interventions and targeted public policies. Identification of clusters with high vaccination rates in the western region of the state, in contrast to low rates in the north and in the Itajaí Valley, suggests the need to investigate and address the specific causes of these regional disparities. In order to mitigate these inequalities, it is important to monitor vaccination coverage according to social, economic and spatial dimensions. 

It is important for information generated by monitoring to inform technical and financial support for municipalities, strengthening their infrastructure and professionals working in the Brazilian National Health System and expanding access to health services. Local planning actions focused on these municipalities with smaller coverage must be conducted to indicate the specific actions most necessary in the context. These findings of our study reinforce the importance of the active participation of the state government and the implementation of public policies that aim to reduce socioeconomic and regional inequalities in vaccination coverage, ensuring equitable access to vaccination against COVID-19 for the entire population of Santa Catarina.

## Data Availability

The vaccination database can be accessed at the following repository: https://github.com/covid19br/dados-vacinas. The 2010 Demographic Census population data can be accessed at: https://sidra.ibge.gov.br/tabela/1378 and the 2022 Demographic Census population data at: https://sidra.ibge.gov.br/tabela/9514.

## Supplementary Material

Supplementary Figure 1

Supplementary Figure 2

Supplementary Figure 3

## References

[1] Mohammed I, Nauman A, Paul P, Ganesan S, Chen K-H, Jalil SMS (2022). The efficacy and effectiveness of the COVID-19 vaccines in reducing infection, severity, hospitalization, and mortality: a systematic review. Hum Vaccin Immunother.

[2] Polack FP, Thomas SJ, Kitchin N, Absalon J, Gurtman A, Lockhart S (2020). Safety and Efficacy of the BNT162b2 mRNA Covid-19 Vaccine. N Engl J Med.

[3] Bar-On YM, Goldberg Y, Mandel M, Bodenheimer O, Freedman L, Kalkstein N (2021). Protection of BNT162b2 Vaccine Booster against Covid-19 in Israel. N Engl J Med.

[4] World Health Organization (2024). WHO COVID-19 dashboard [Internet].

[5] United Nations Development Programme (2021). Global Dashboard for Vaccine Equity [Internet].

[6] Fonseca EM, Nattrass N, Lazaro LLB, Bastos FI (2021). Political discourse, denialism and leadership failure in Brazil’s response to COVID-19. Glob Public Health.

[7] Brasil (2024). Cobertura vacinal covid-19.

[8] Boing AF, Boing AC, Barberia L, Borges ME, Subramanian SV (2023). Uncovering inequities in Covid-19 vaccine coverage for adults and elderly in Brazil: A multilevel study of 2021-2022 data. Vaccine.

[9] Paim J, Travassos C, Almeida C, Bahia L, Macinko J (2011). The Brazilian health system: history, advances, and challenges. Lancet.

[10] Boing AF, Boing AC, Veras MA, Lacerda JT, Silva RLP, Barbato PR (2020). Area-level inequalities in Covid-19 outcomes in Brazil in 2020 and 2021: An analysis of 1,894,165 severe Covid-19 cases. Prev Med.

[11] Brasil (2024). Cobertura Vacinal Covid-19.

[12] Boing AF, Boing AC, Barberia L, Borges ME, Subramanian SV (2023). The Brazilian vaccine divide: How some municipalities were left behind in the Covid-19 vaccine coverage. PLOS Glob Public Health.

[13] Holanda WTG, Oliveira SB, Sanchez MN (2022). Aspectos diferenciais do acesso e qualidade da atenção primária à saúde no alcance da cobertura vacinal de influenza. Cien Saude Colet.

[14] Darrudi A, Khoonsari MHK, Tajvar M (2022). Challenges to Achieving Universal Health Coverage Throughout the World: A Systematic Review. J Prev Med Public Health.

[15] Martins JRT, Alexandre BGP, Oliveira VC, Viegas SMF (2018). Educação permanente em sala de vacina: qual a realidade?. Rev Bras Enferm.

[16] Arsenault C, Lewis TP, Kapoor NR, Okiro EA, Leslie HH, Armeni P (2024). Health system quality and COVID-19 vaccination: a cross-sectional analysis in 14 countries. Lancet Glob Health.

[17] Arce JSS, Warren SS, Meriggi NF, Scacco A, McMurry N, Voors M (2021). COVID-19 vaccine acceptance and hesitancy in low- and middle-income countries. Nat Med.

[18] Kamis C, Stolte A, West JS, Fishman SH, Brown T, Brown T (2021). Overcrowding and COVID-19 mortality across U.S. counties: Are disparities growing over time?. SSM Popul Health.

[19] Liu Y, Wang K, Yang L, He D (2022). Regional heterogeneity of in-hospital mortality of COVID-19 in Brazil. Infect Dis Model.

[20] Paschoalotto MAC, Costa EPPA, Almeida SV, Cima J, Costa JG, Santos JV (2021). Running away from the jab: factors associated with COVID-19 vaccine hesitancy in Brazil. Rev Saude Publica.

[21] Mondal P, Sinharoy A, Su L (2021). Sociodemographic predictors of COVID-19 vaccine acceptance: a nationwide US-based survey study. Public Health.

[22] Pimentel SM, Avila MAG, Prata RA, Nunes HRC, Silva JB (2022). Associação entre letramento em saúde, ameaça pela COVID-19 e intenção vacinal de adolescentes brasileiros. Rev. Lat-Am Enfermagem.

[23] Domingues CMAS, Fantinato FFST, Duarte E, Garcia LP (2019). Vacina Brasil e estratégias de formação e desenvolvimento em imunizações. Epidemiol.

[24] Argeros G, Hoffman JL, Dove N (2023). An Exploratory Ecological Study between COVID-19 Vaccination Rate and Racial/Ethnic and Socioeconomic Status Neighborhood Conditions in Michigan. COVID 2023.

[25] Hernandez I, Dickson S, Tang S, Gabriel N, Berenbrok LA, Guo J (2022). Disparities in distribution of COVID-19 vaccines across US counties: A geographic information system–based cross-sectional study. PLoS Med.

[26] Figueiredo AM, Massuda A, Fernandez M, Medeiros  AH, Carvalho M (2024). Imunização contra covid-19 e mortalidade em pacientes hospitalizados: coorte retrospectiva. Rev Saude Publica.

[27] Bastos LSL, Aguilar S, Rache B, Maçaira P, Baião F, Cerbino-Neto J (2022). Primary healthcare protects vulnerable populations from inequity in COVID-19 vaccination: An ecological analysis of nationwide data from Brazil. Lancet Reg Healtyh Am.

[28] Guimarães JRS, Jannuzzi PM (2005). IDH, indicadores sintéticos e suas aplicações em políticas públicas: uma análise crítica. Revista Brasileira de Estudos Urbanos e Regionais.

