# Supplementary figures and images for: Intrastate inequality of COVID-19 vaccination coverage: spatial analysis and socioeconomic, Santa Catarina, 2021-2023
Source: Epidemiol Serv Saude. 2025 Aug 8;34:e20240329. doi: 10.1590/S2237-96222025v34e20240329.en (PMC12342717; doi:10.1590/S2237-96222025v34e20240329.en)

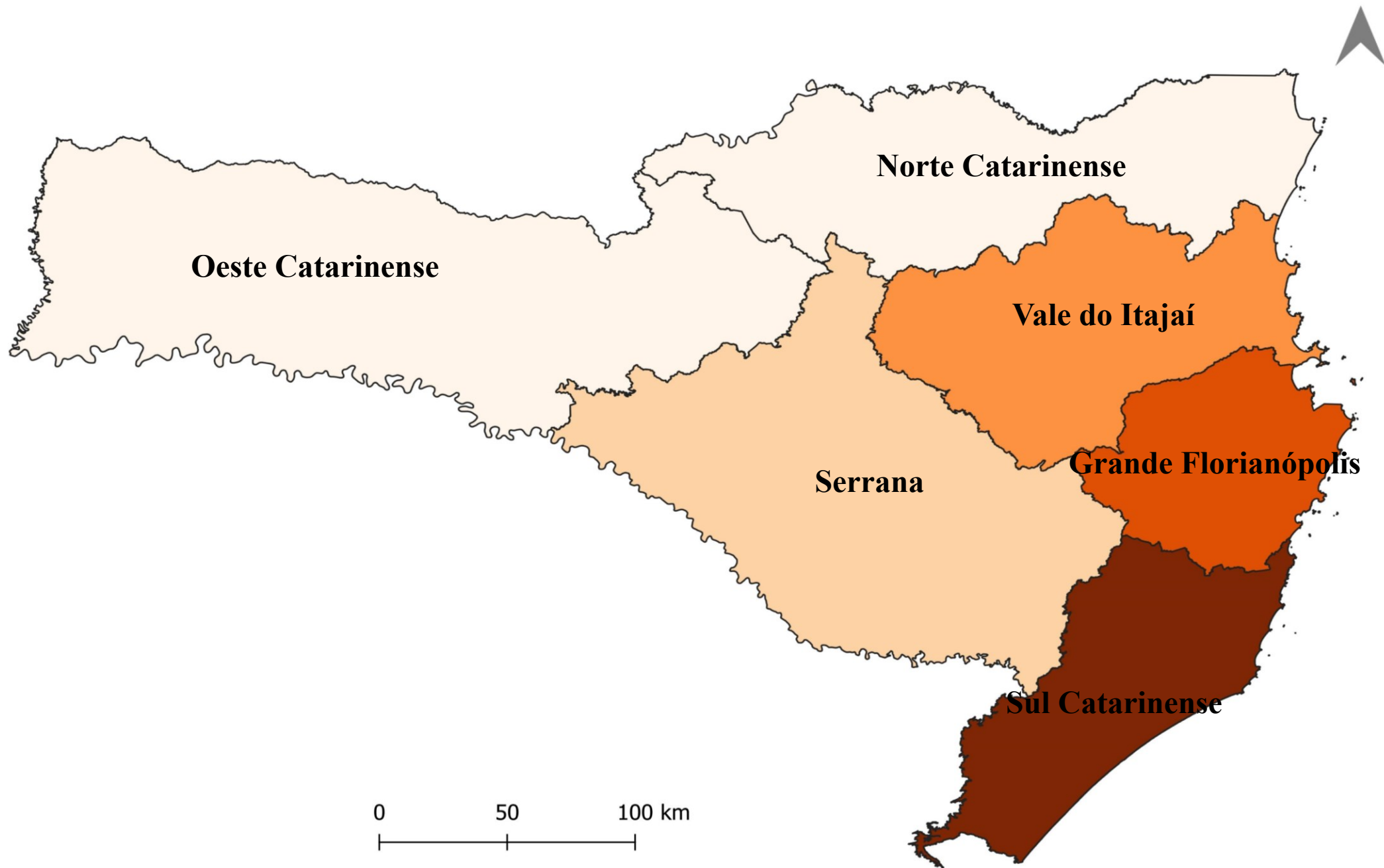

Supplement: Supplementary file 1 [file 2237-9622-ress-34-e20240329-supp01.pdf]

# A1

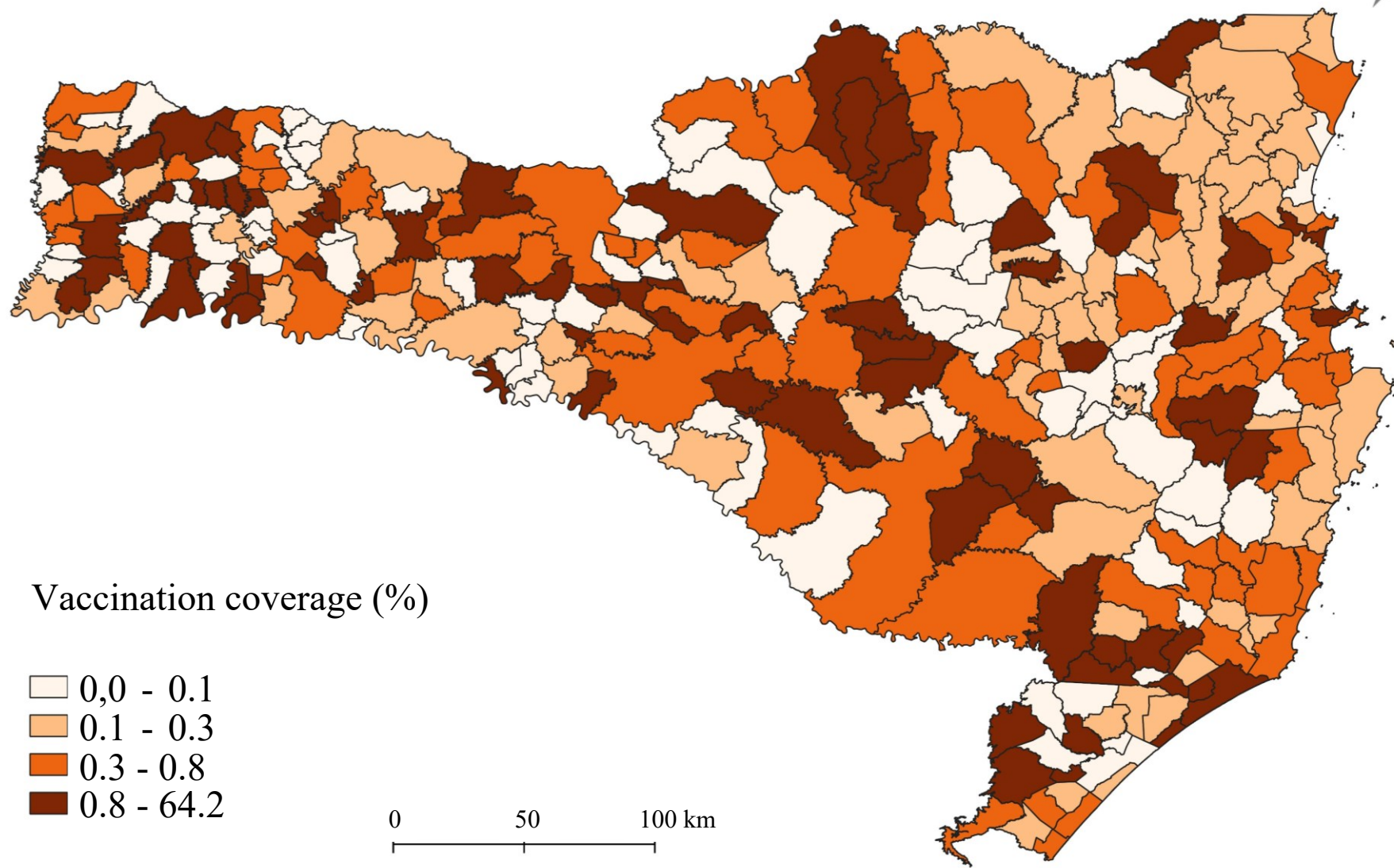

# A2

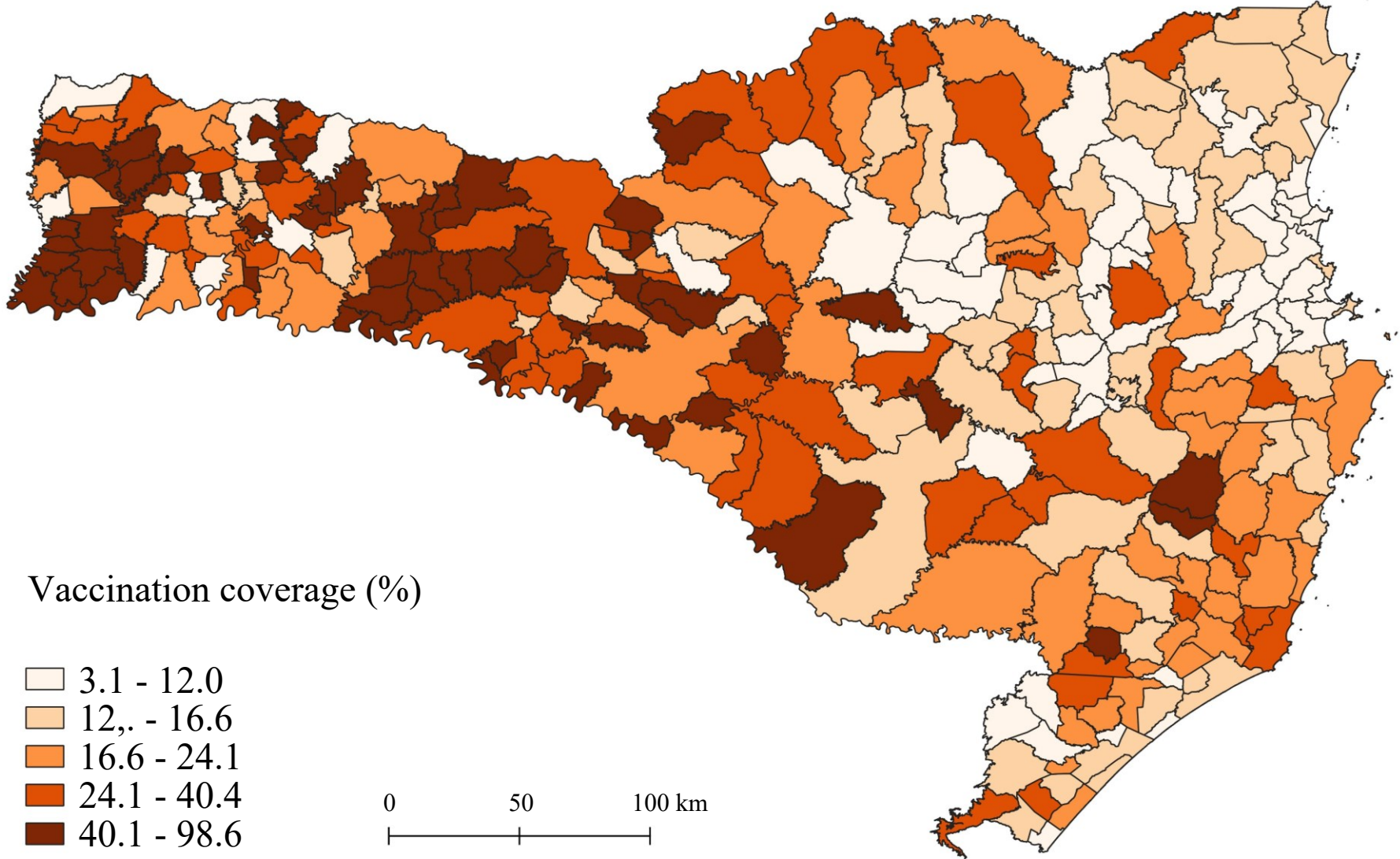

B1

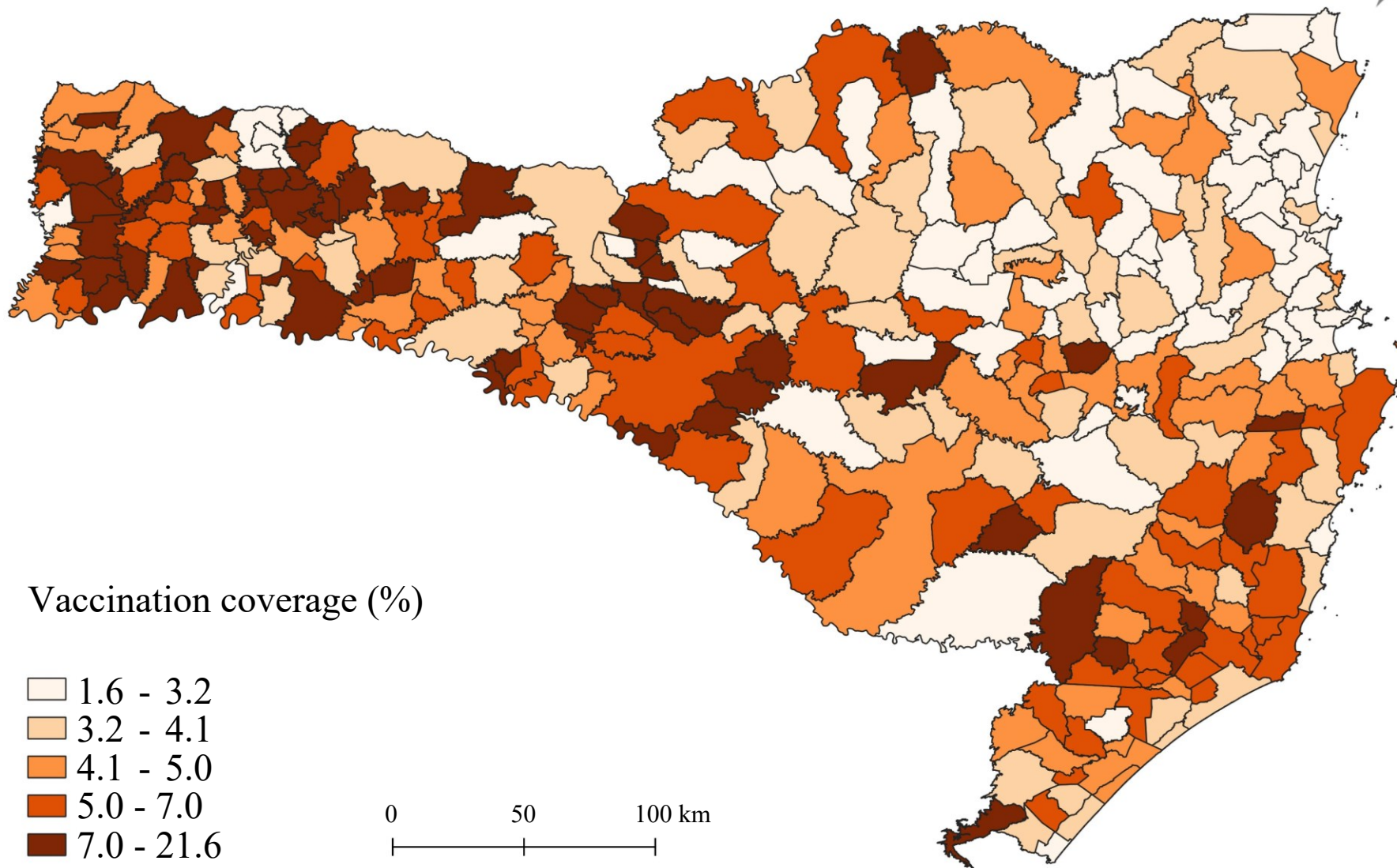

B2

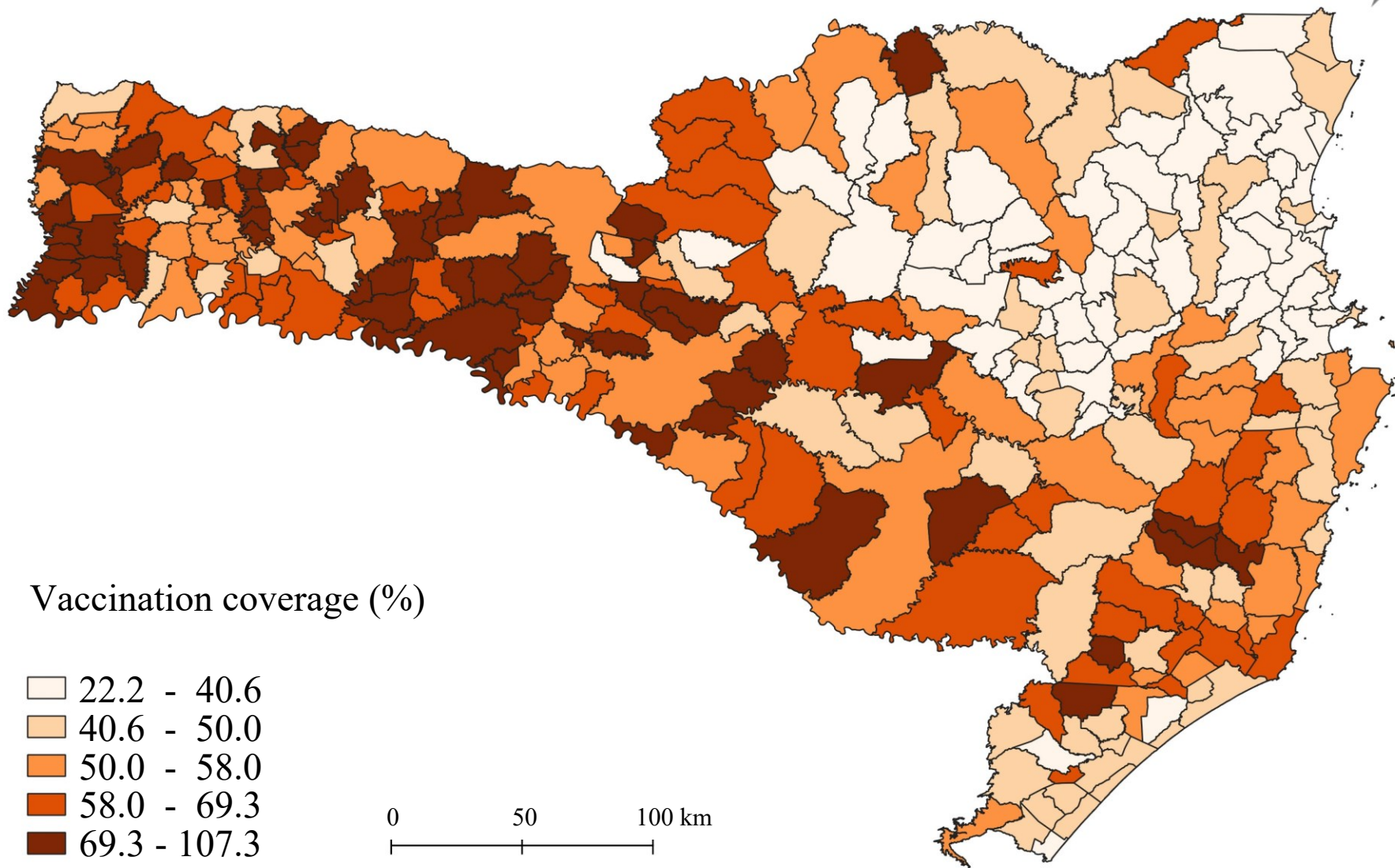

C1

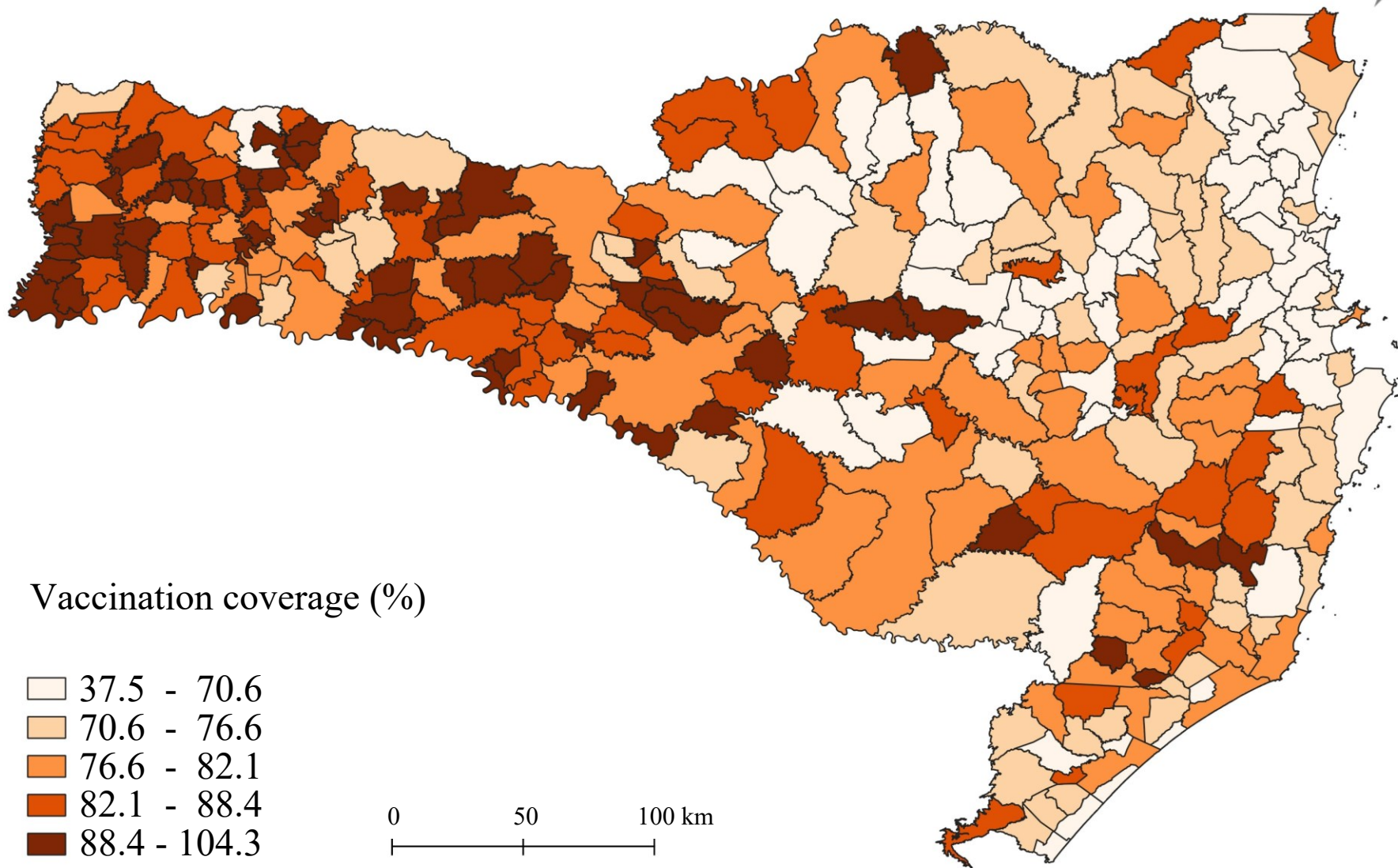

C2

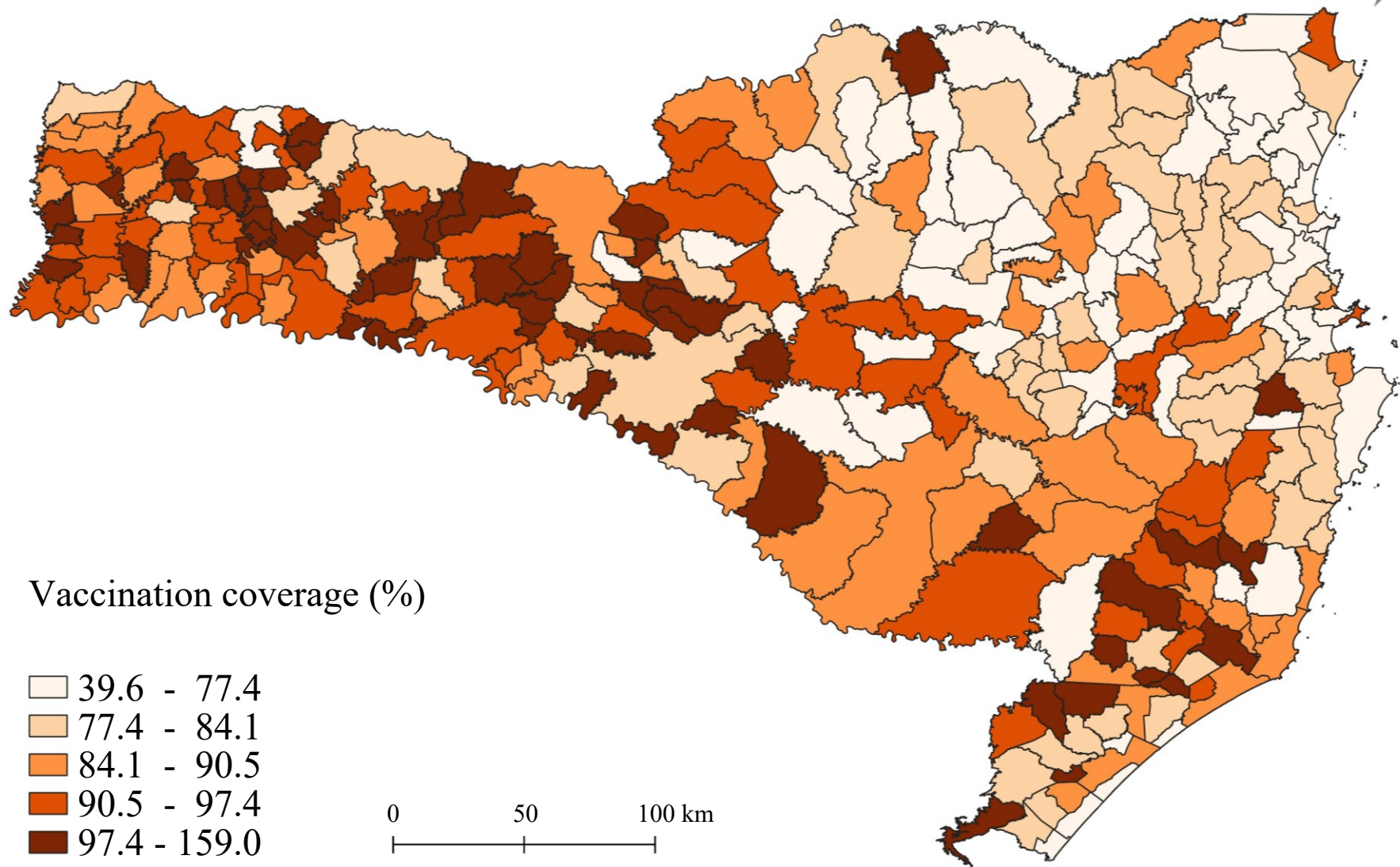

Supplement: Supplementary file 2 [file 2237-9622-ress-34-e20240329-supp02.pdf]

# A1

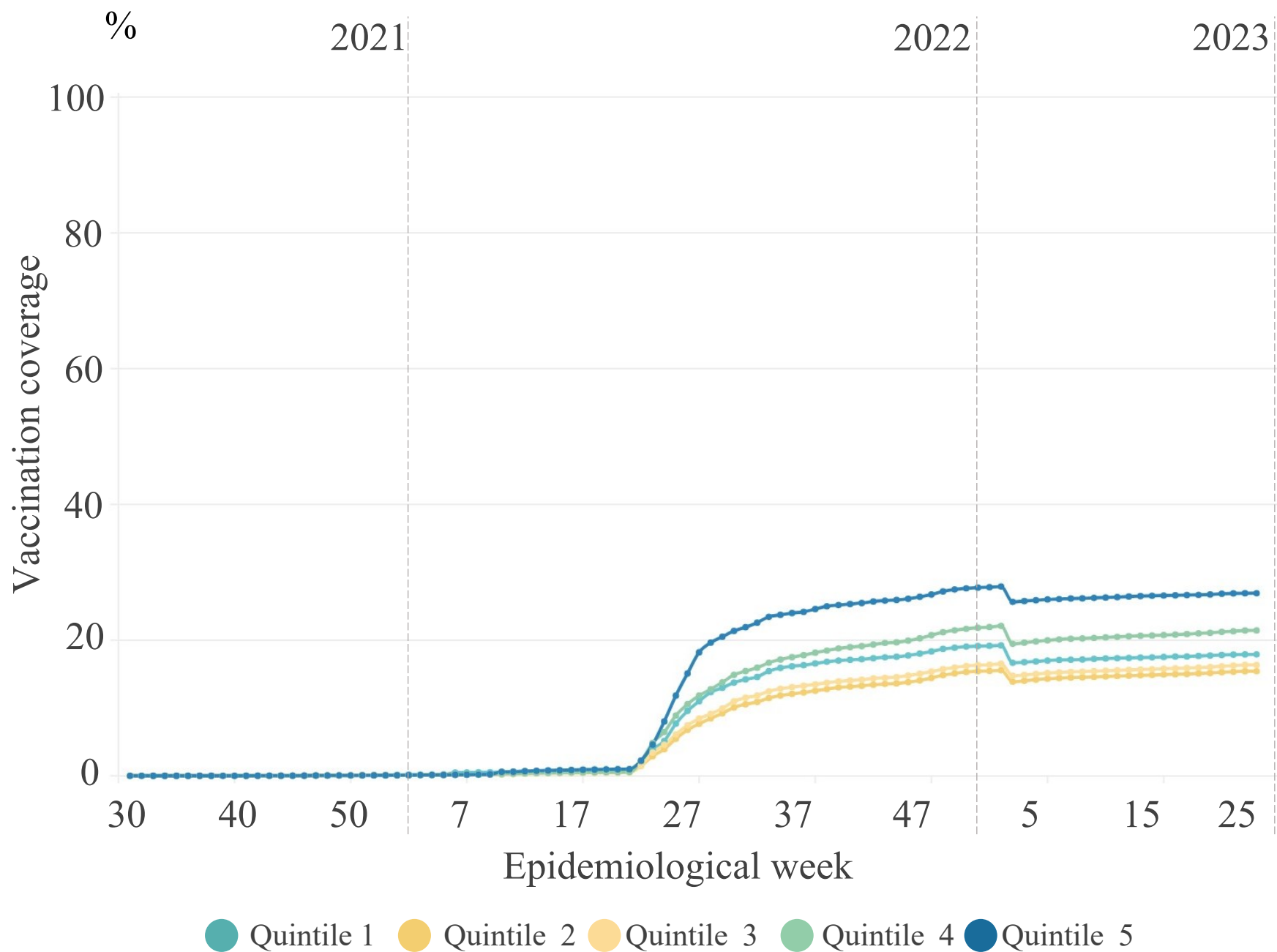

# A2

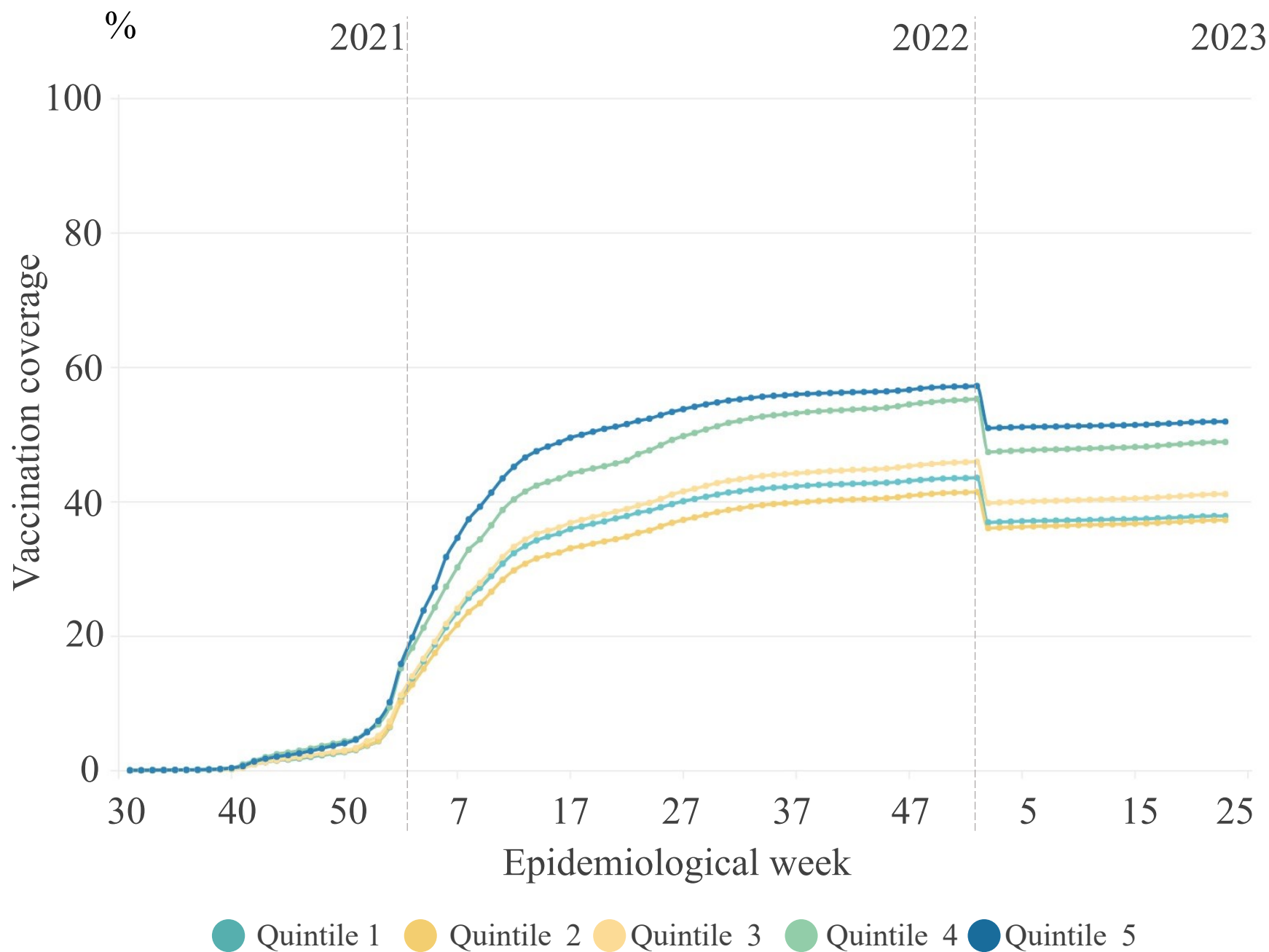

A3

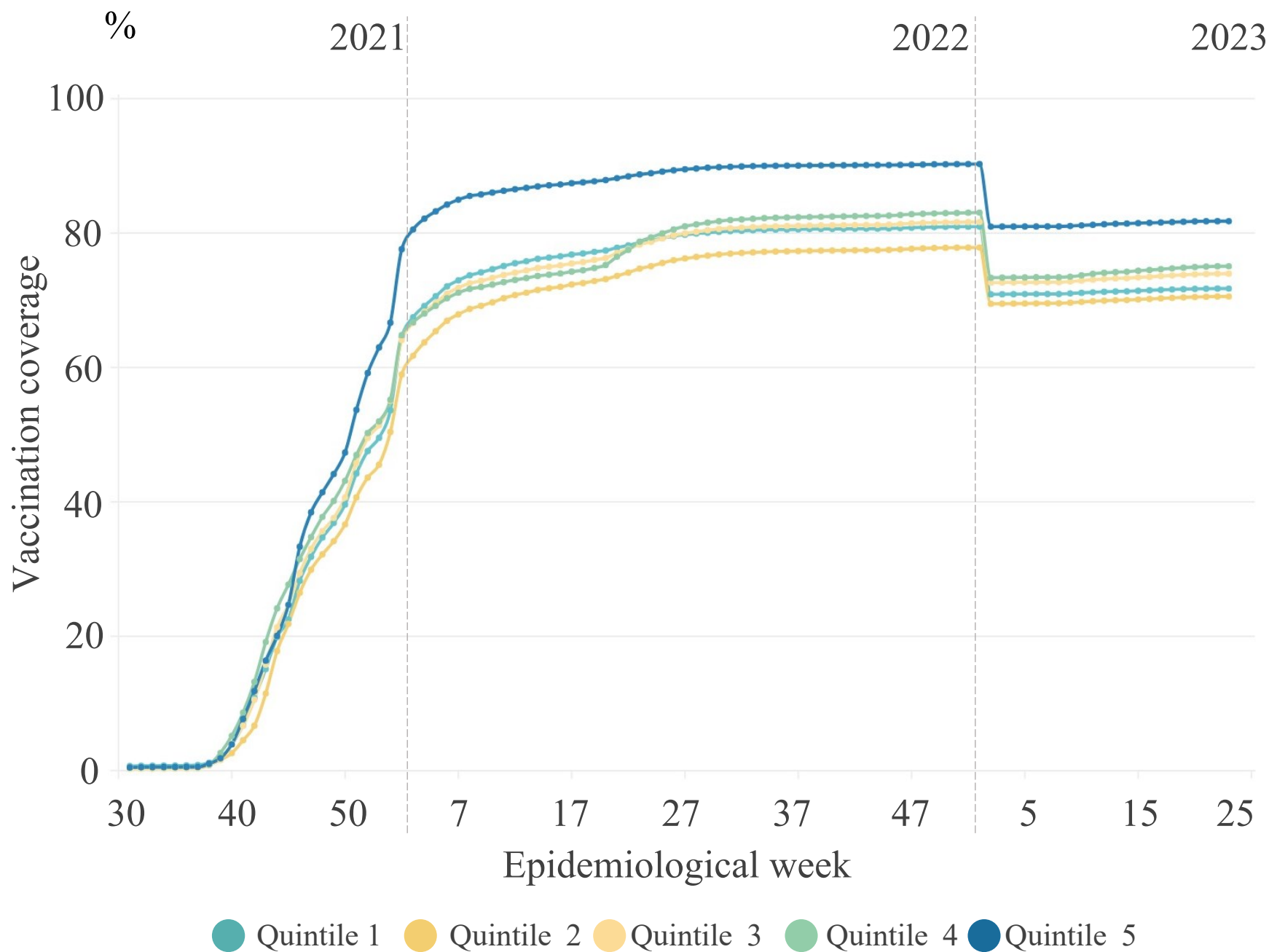

# B1

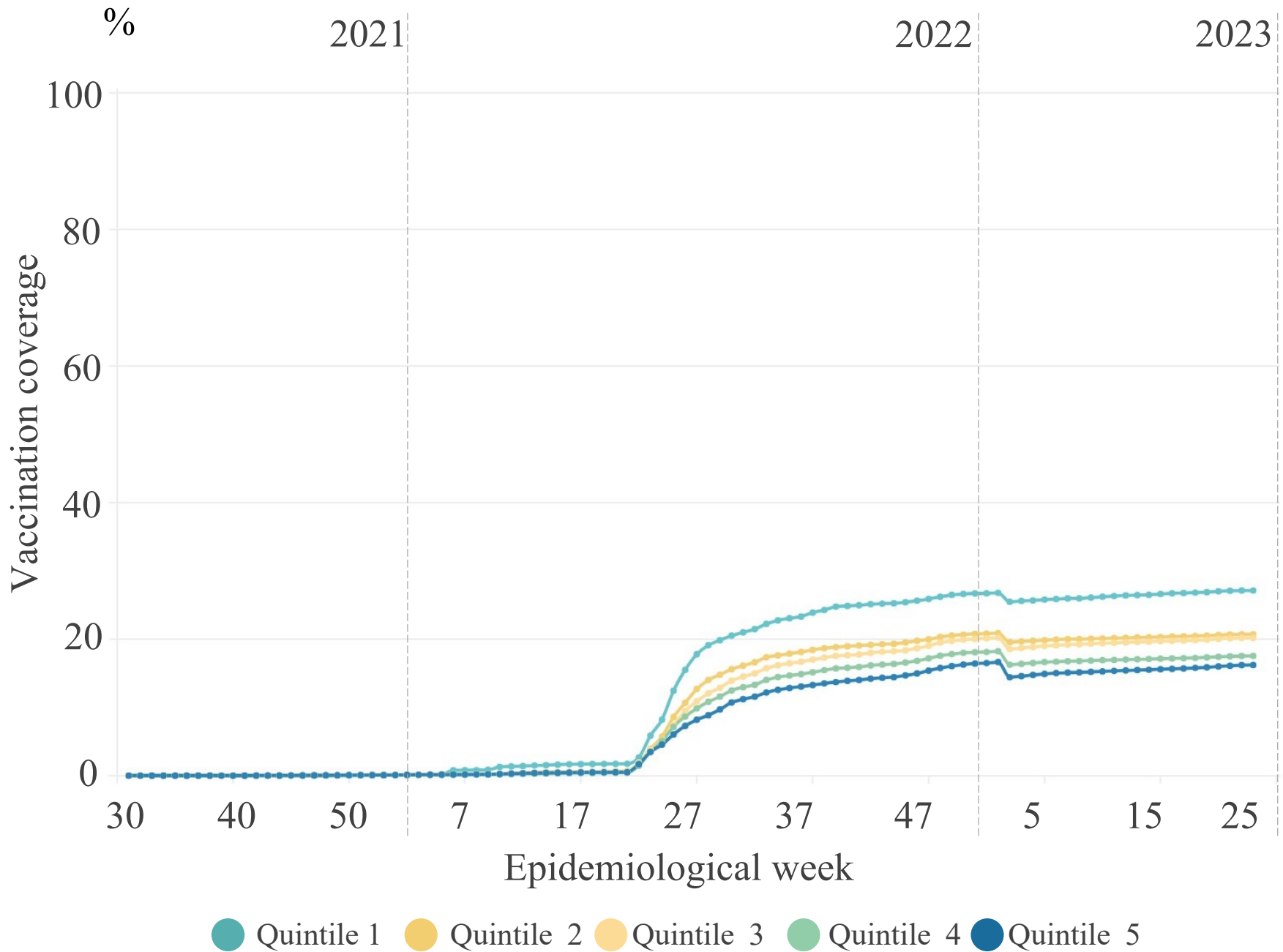

# B2

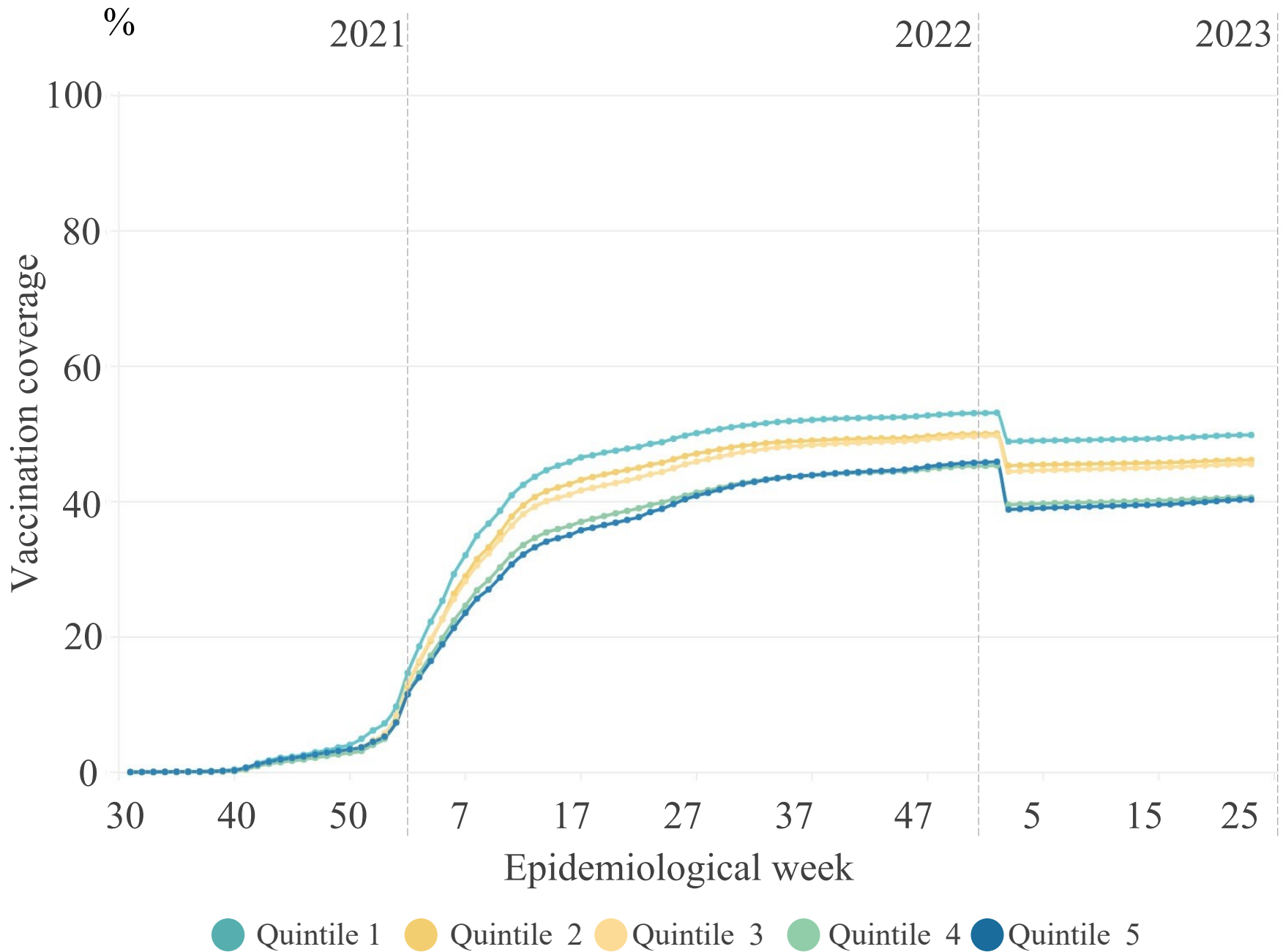

B3

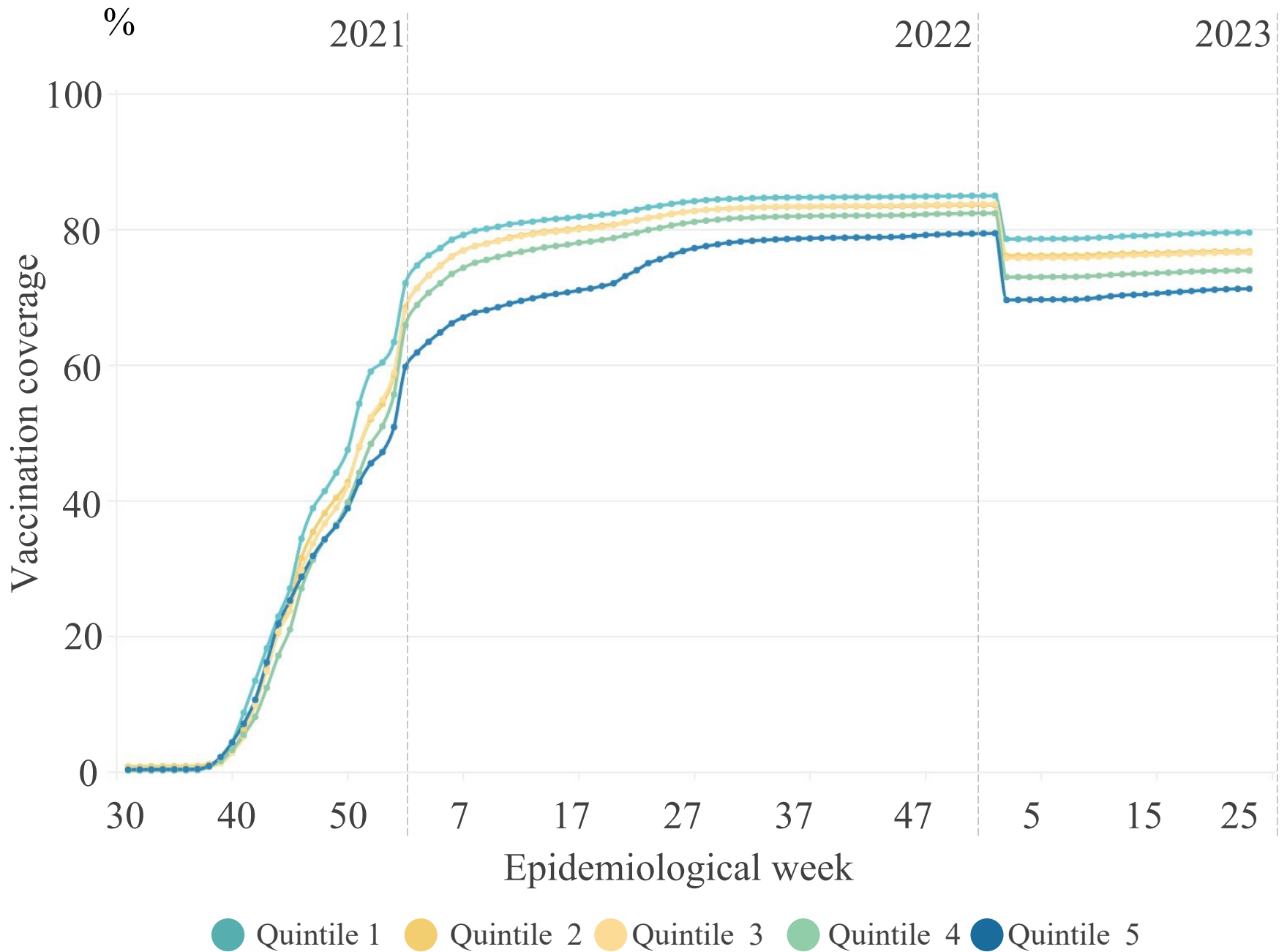

# C1

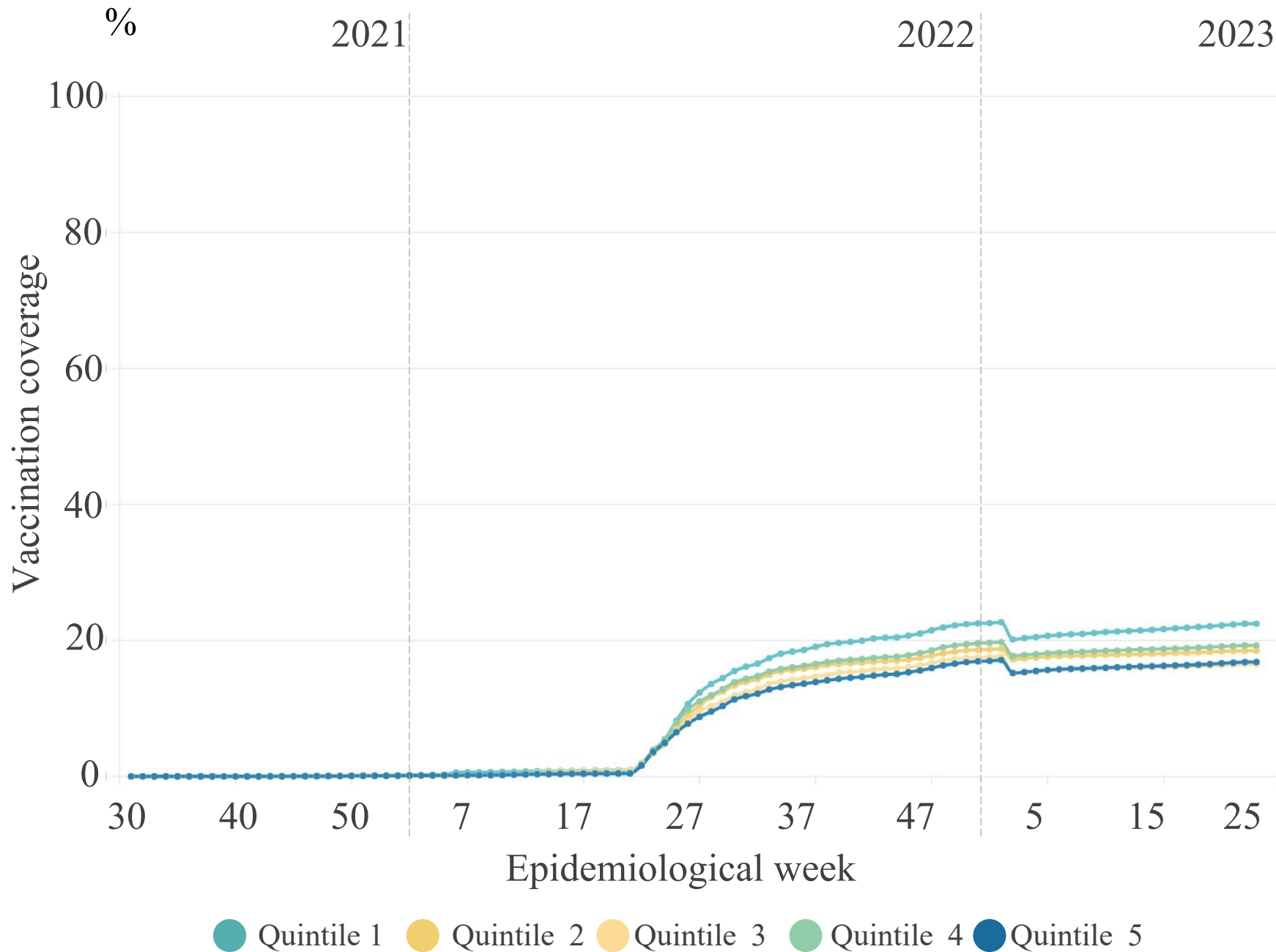

# C2

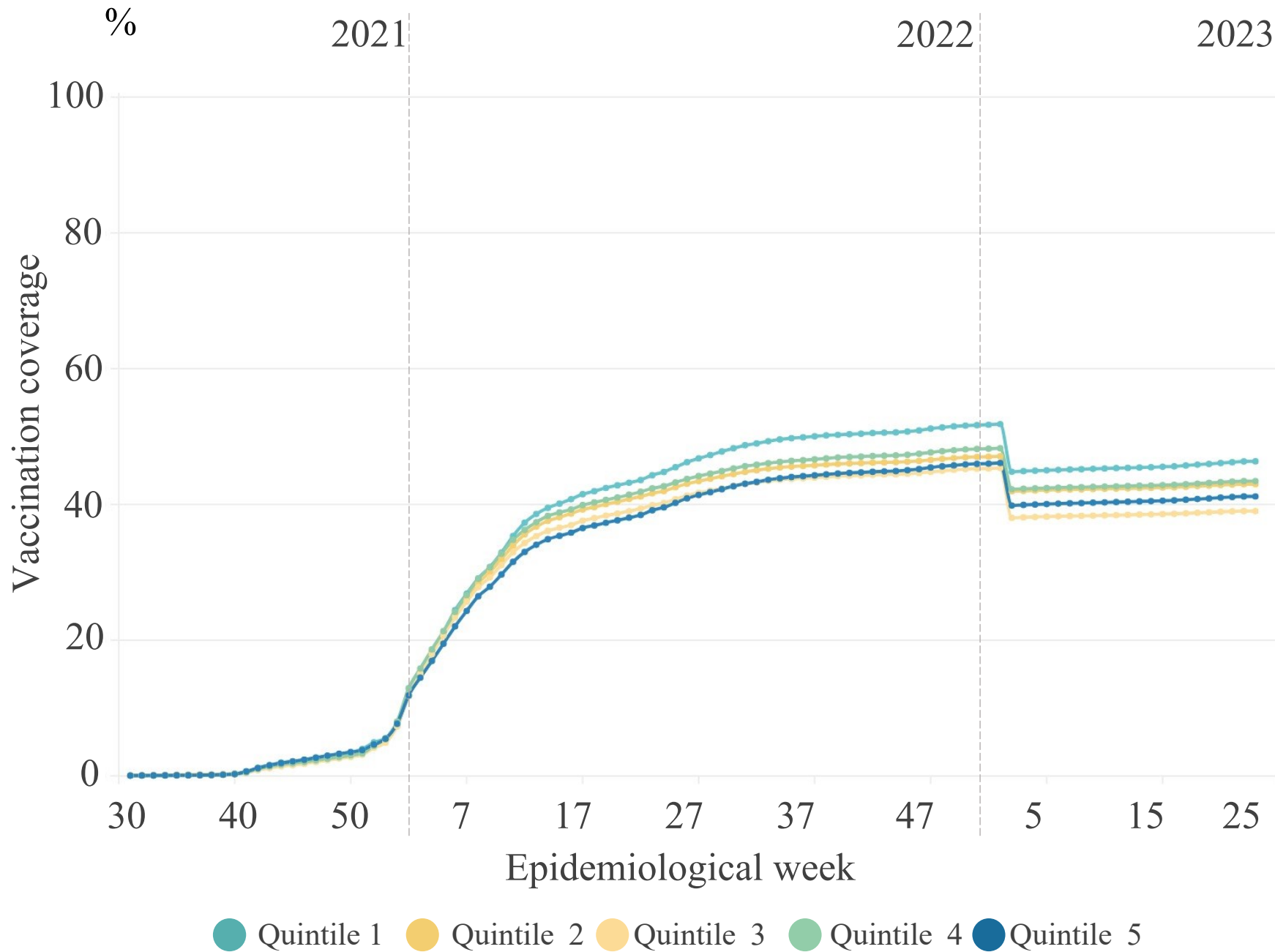

# C3

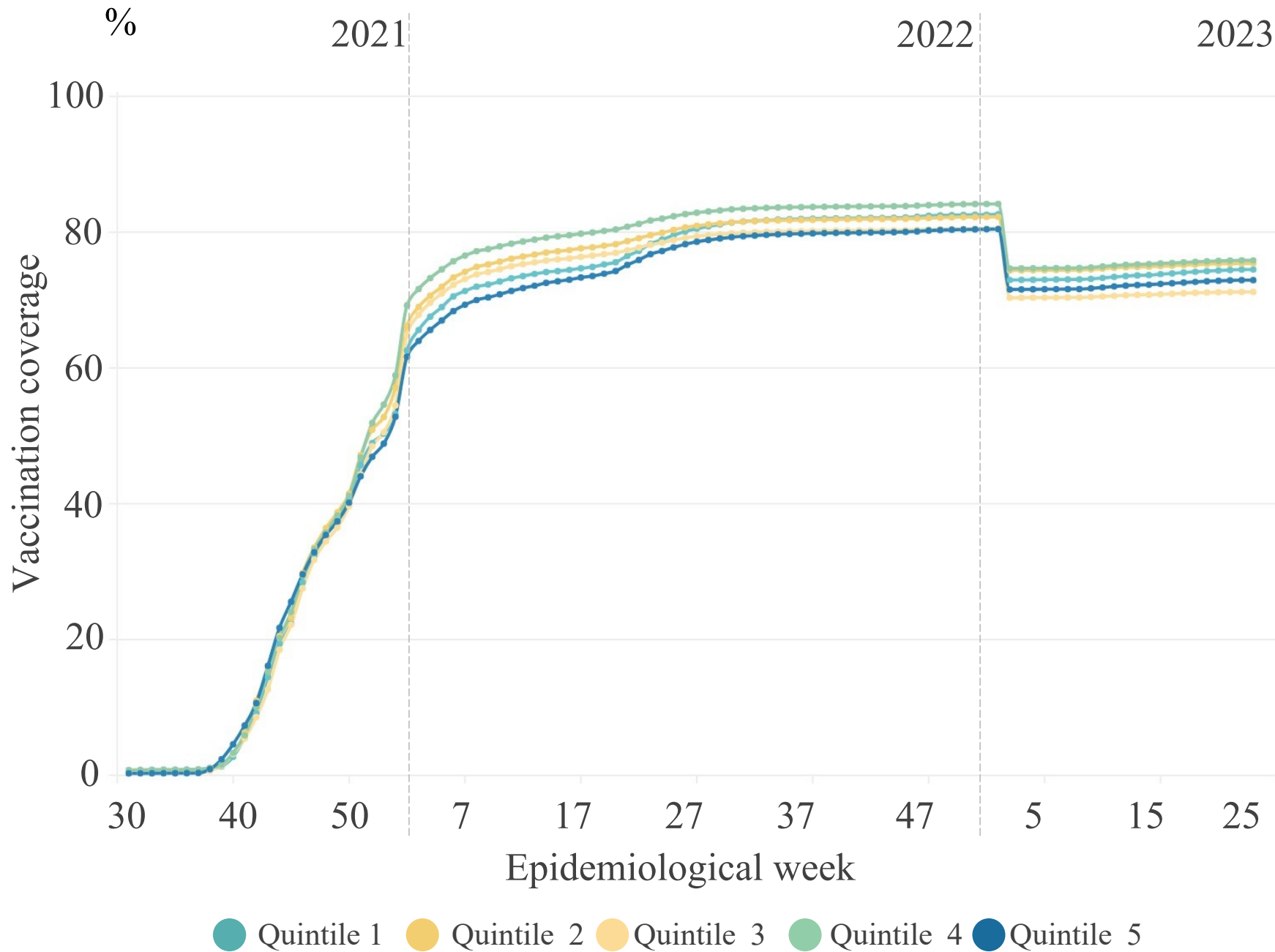

Supplement: Supplementary file 3 [file 2237-9622-ress-34-e20240329-supp03.pdf]

# A1

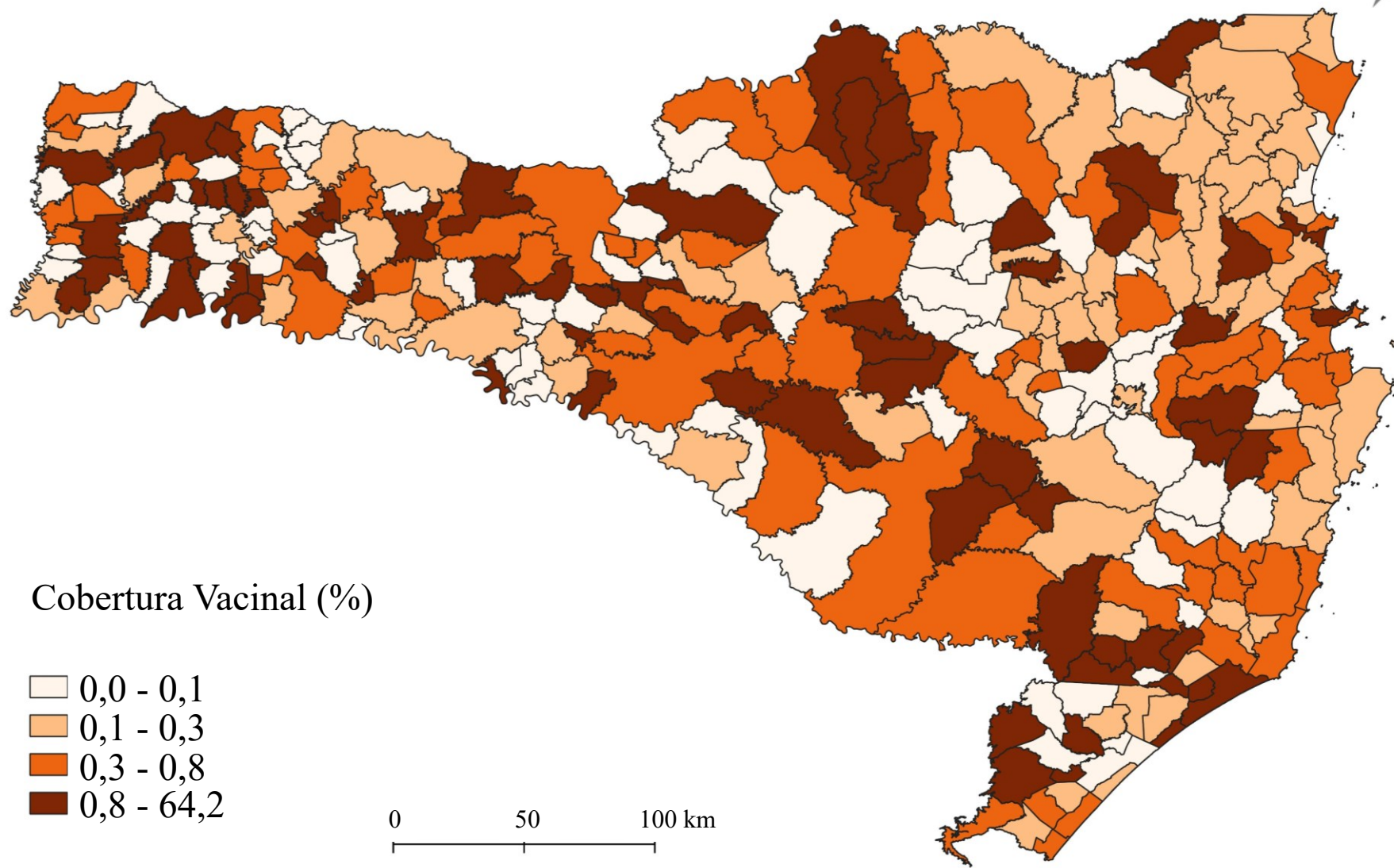

# A2

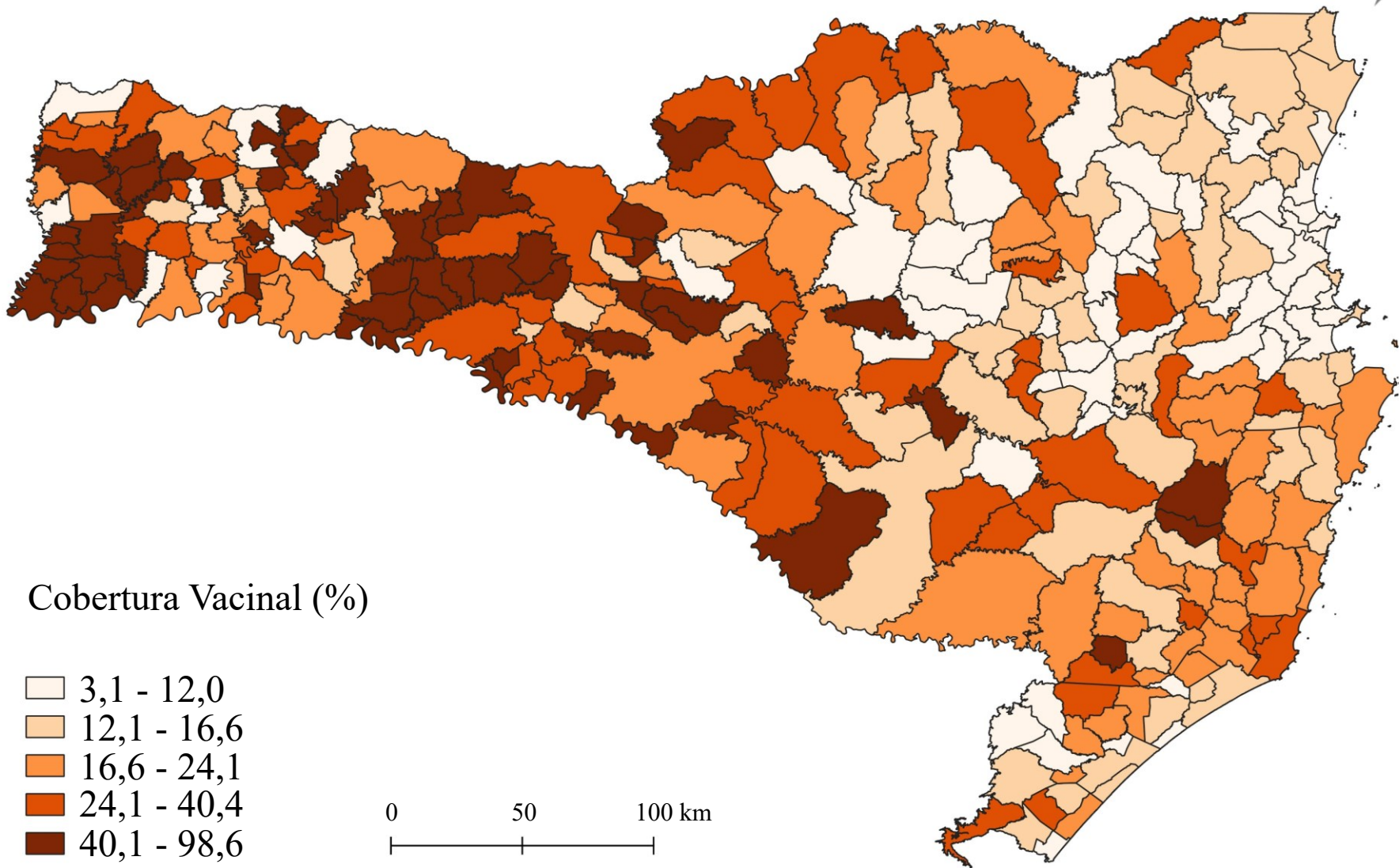

B1

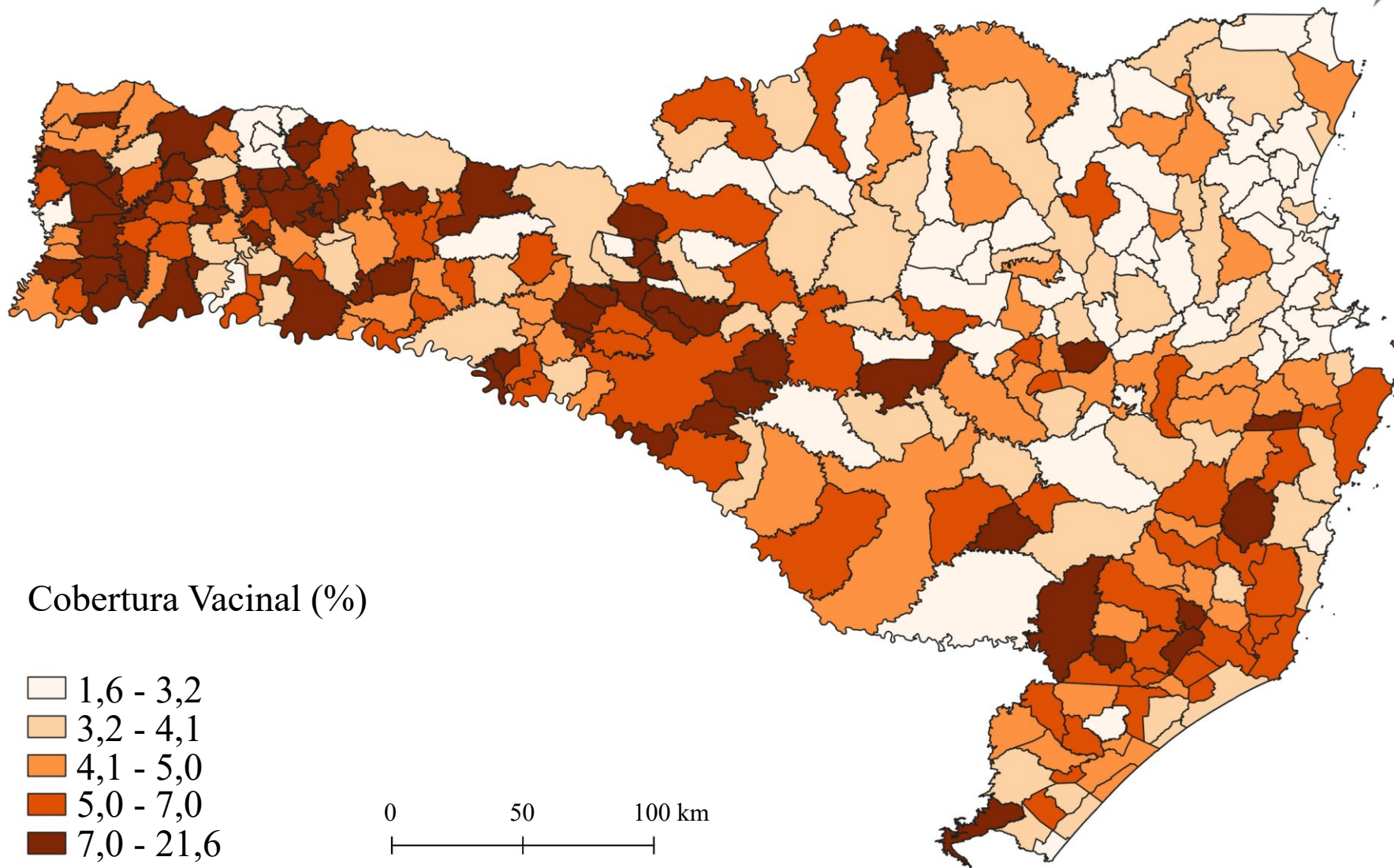

B2

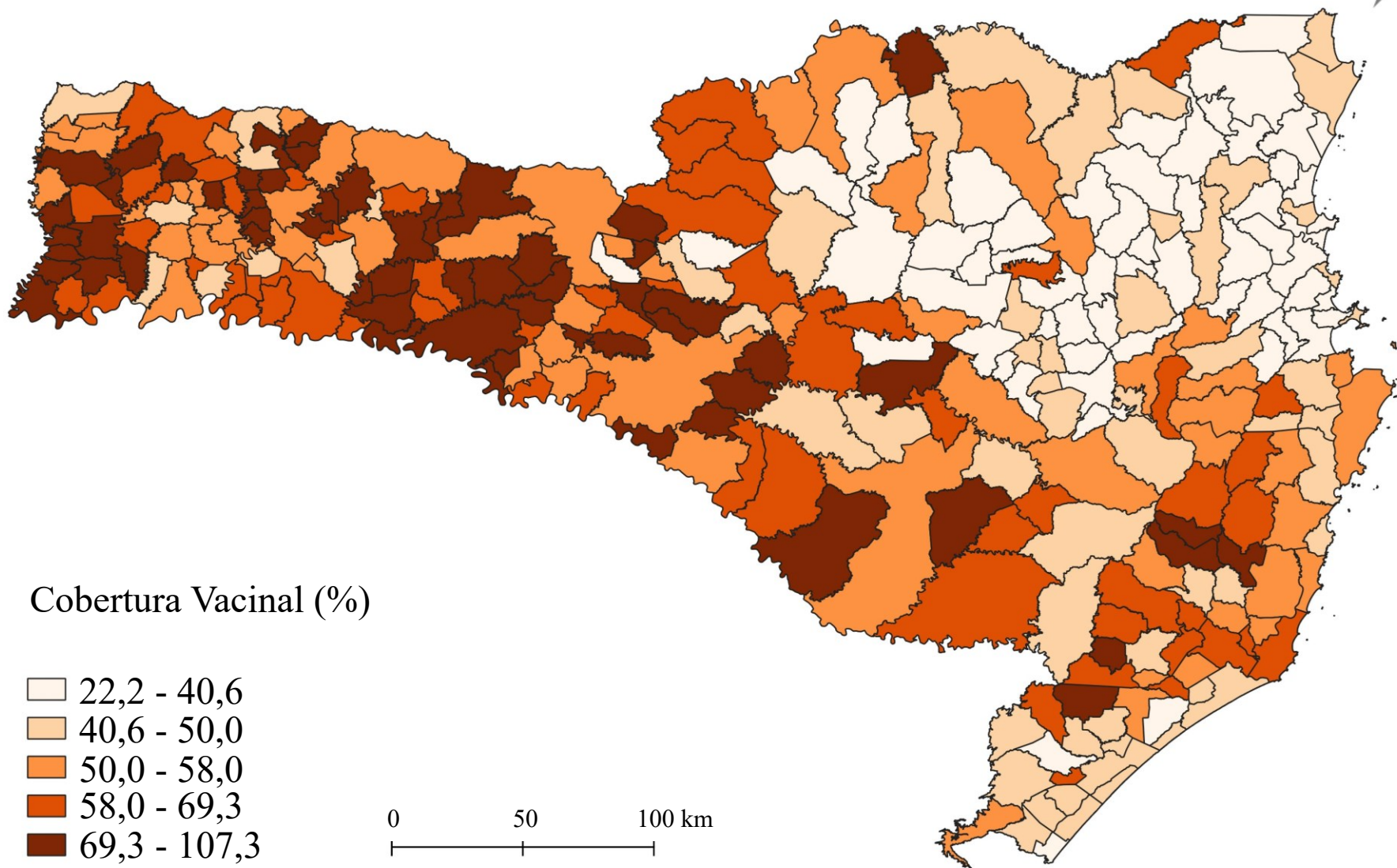

C1

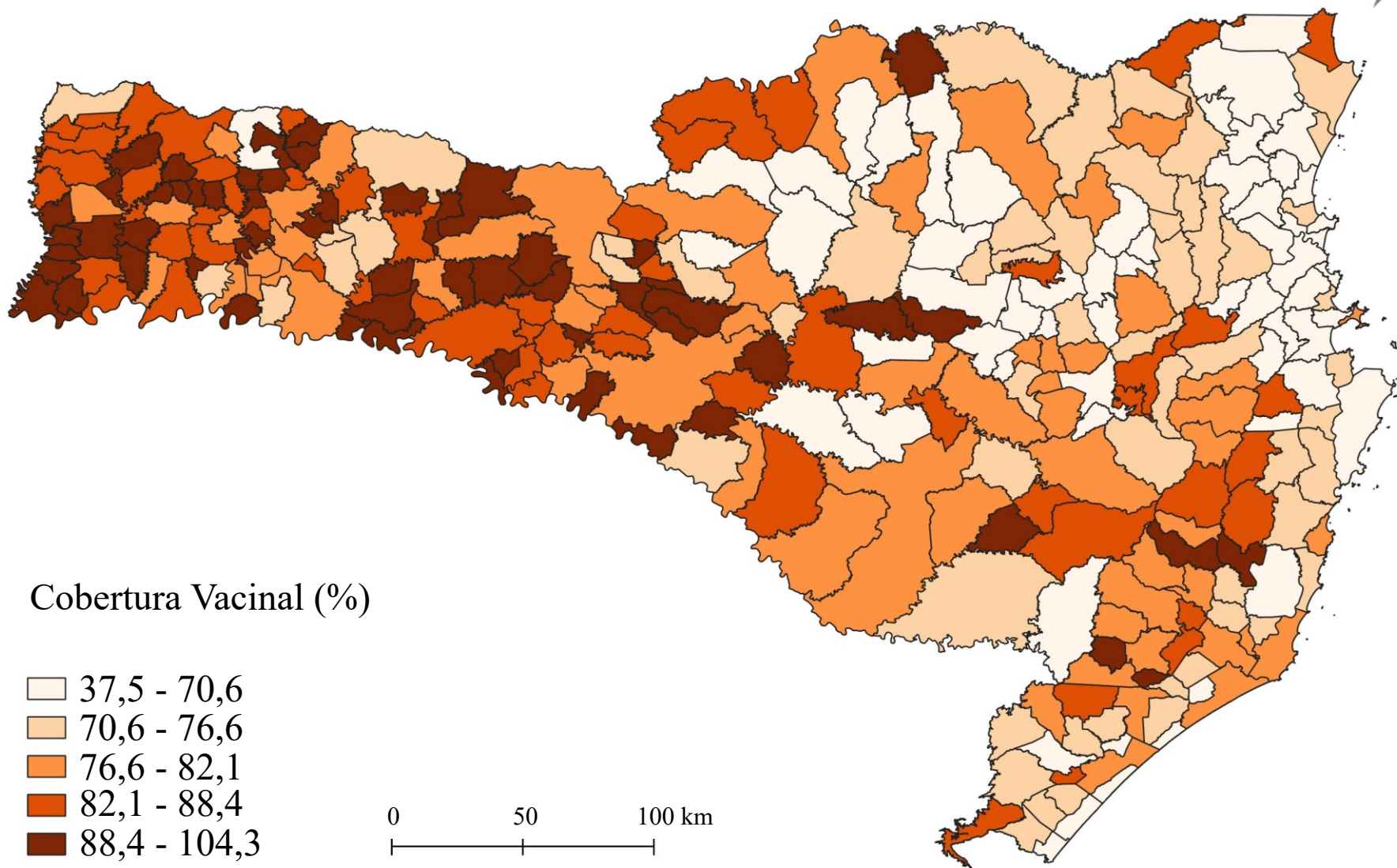

C2

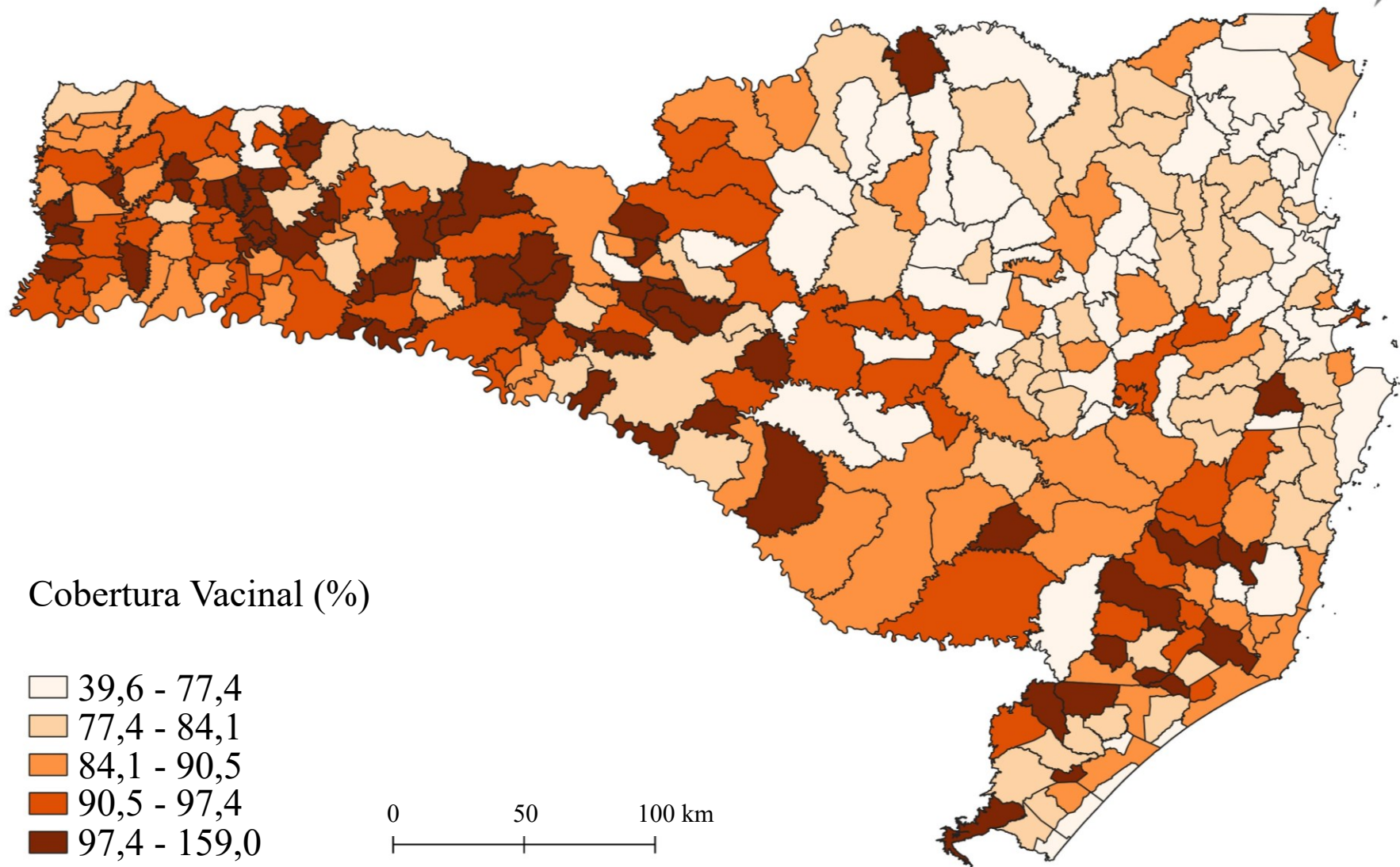

Supplement: Supplementary file 4 [file 2237-9622-ress-34-e20240329-supp02-pt.pdf]

# A1

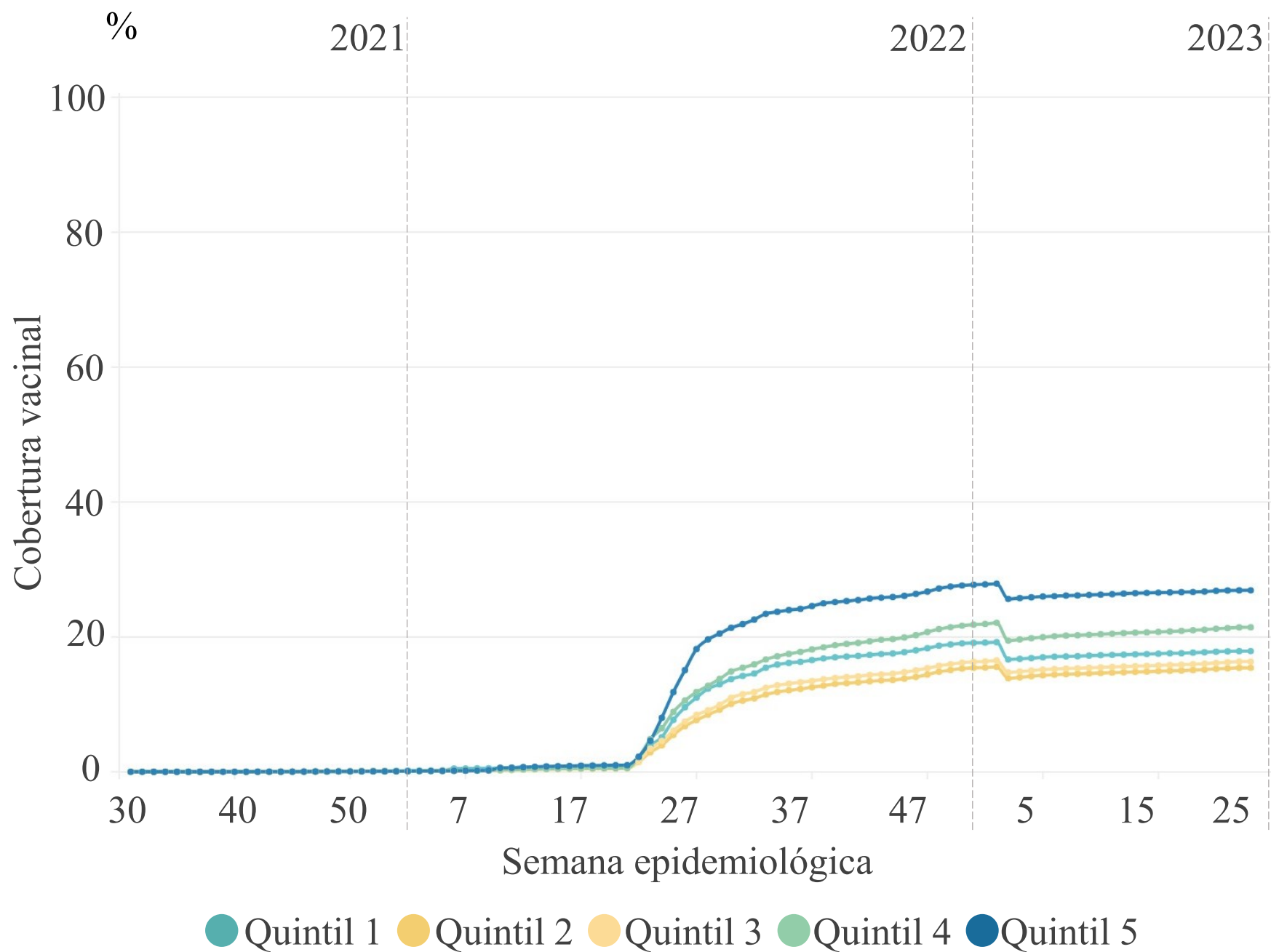

# A2

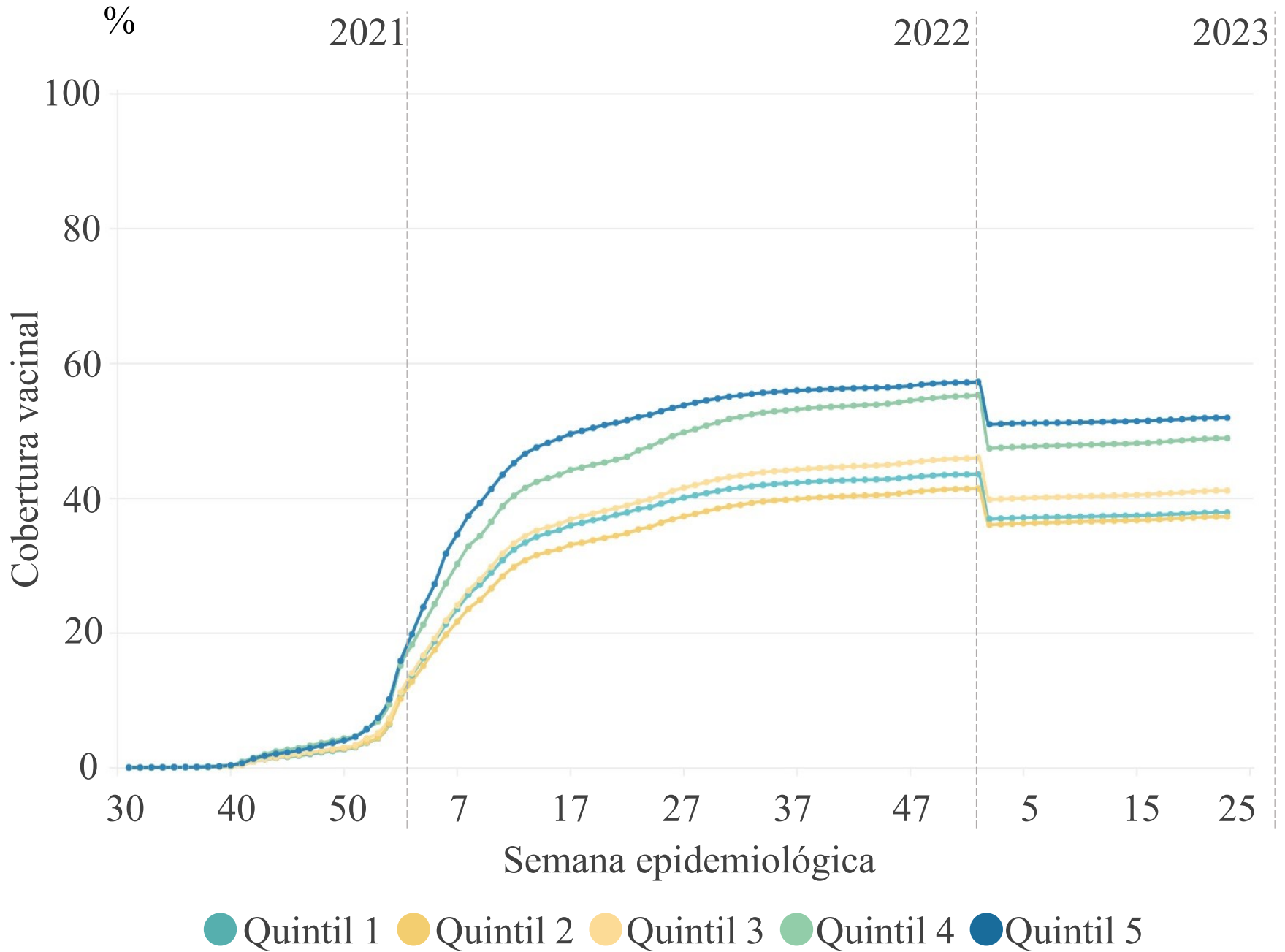

A3

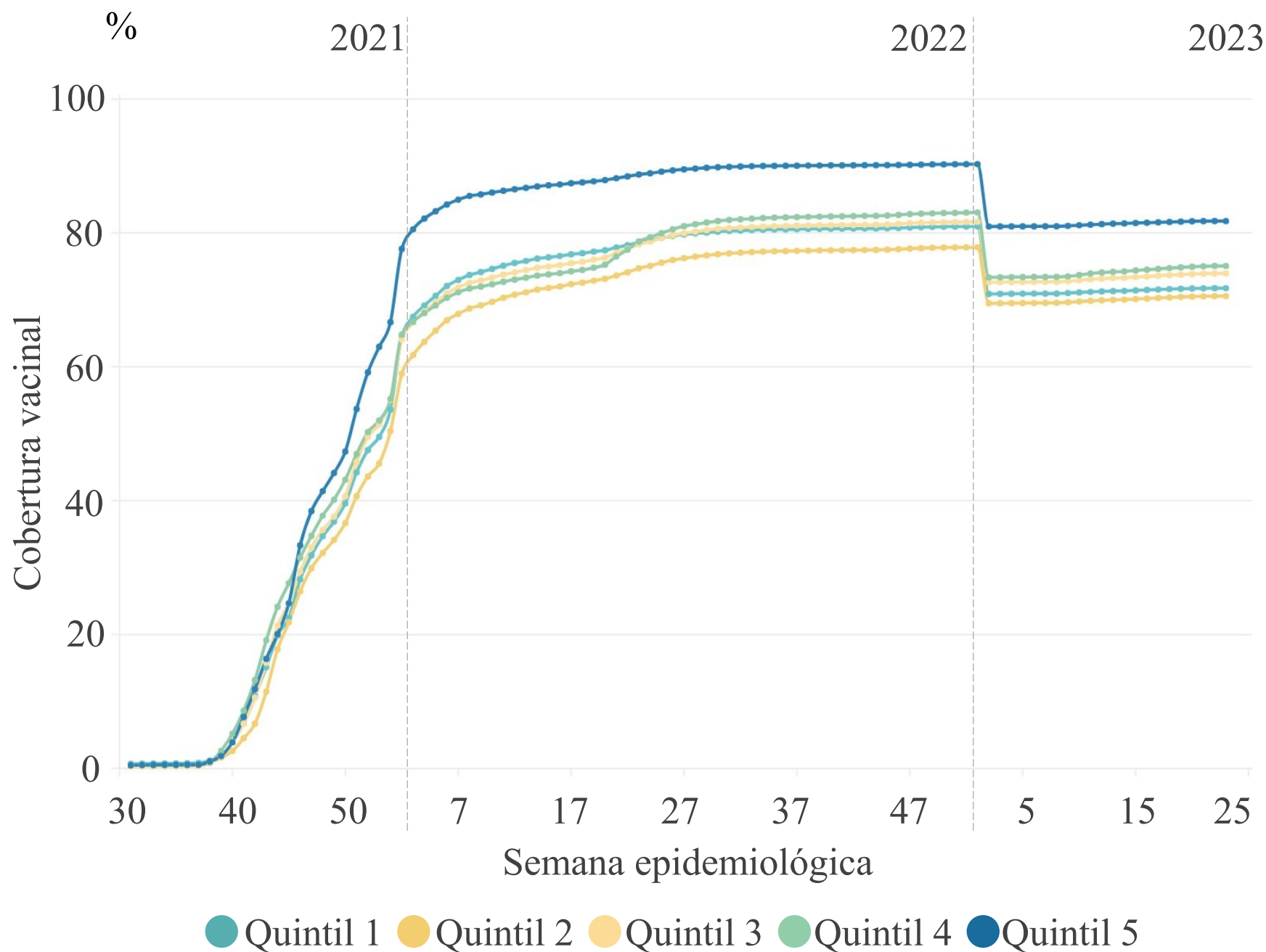

# B1

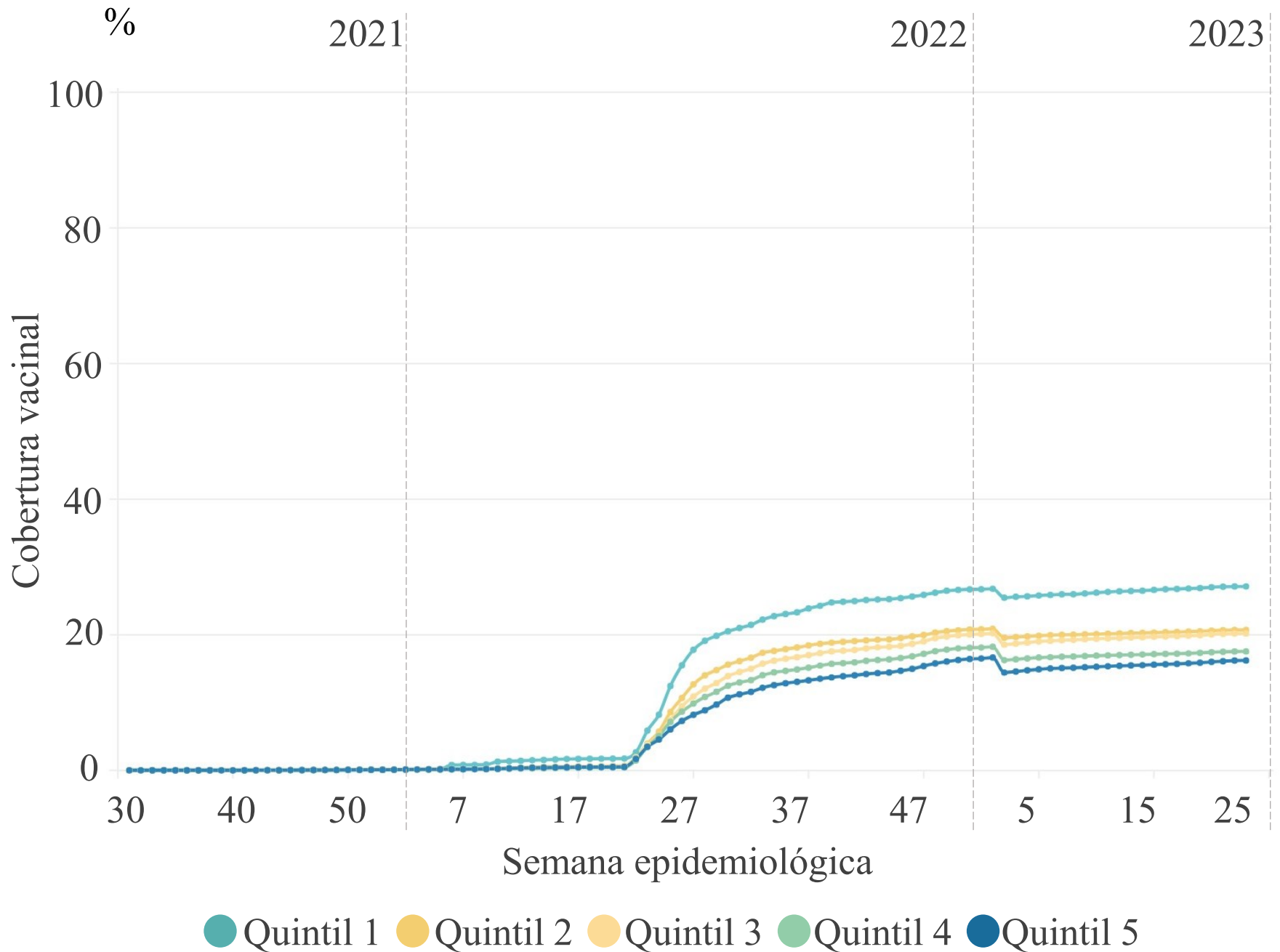

# B2

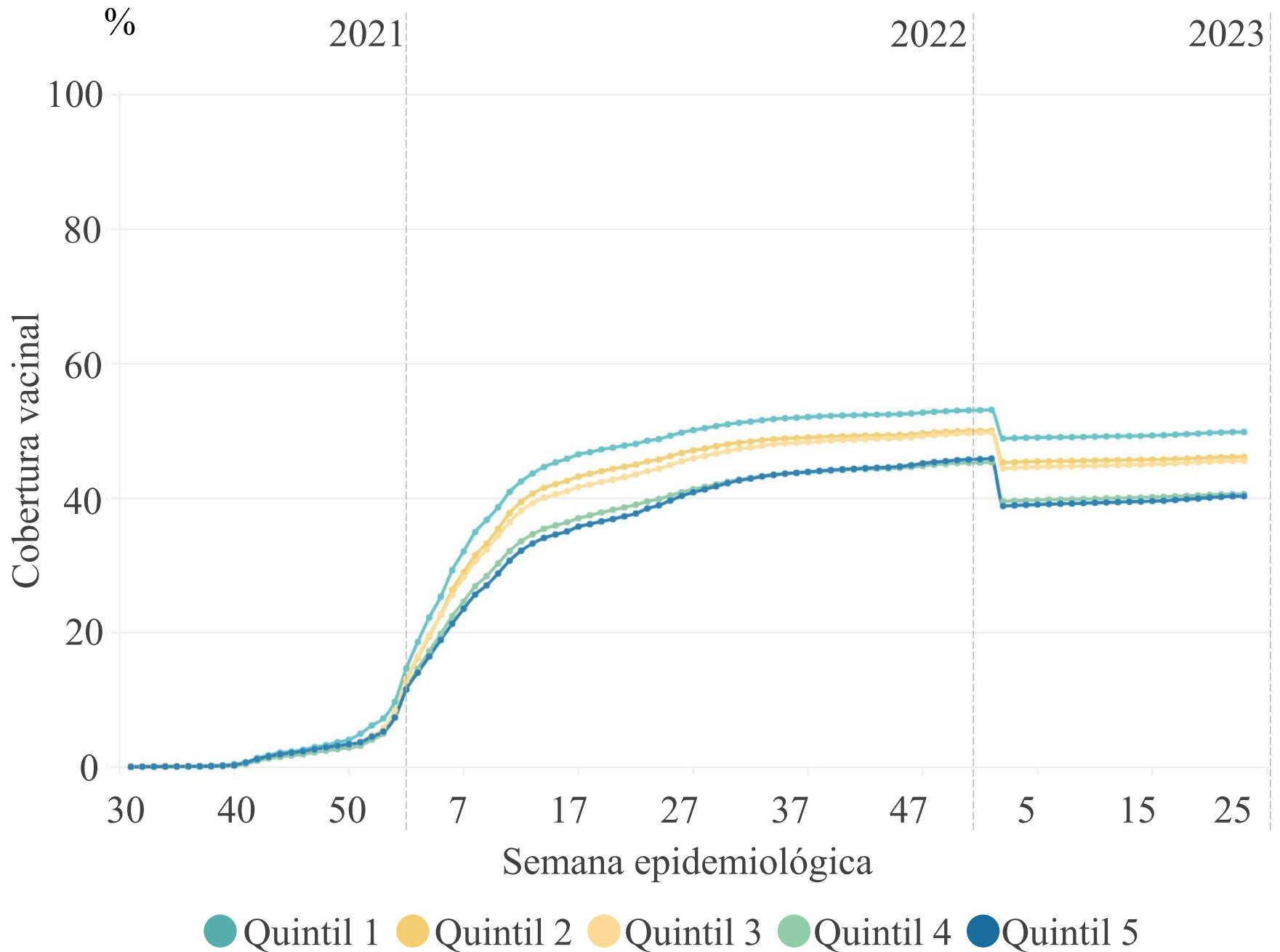

B3

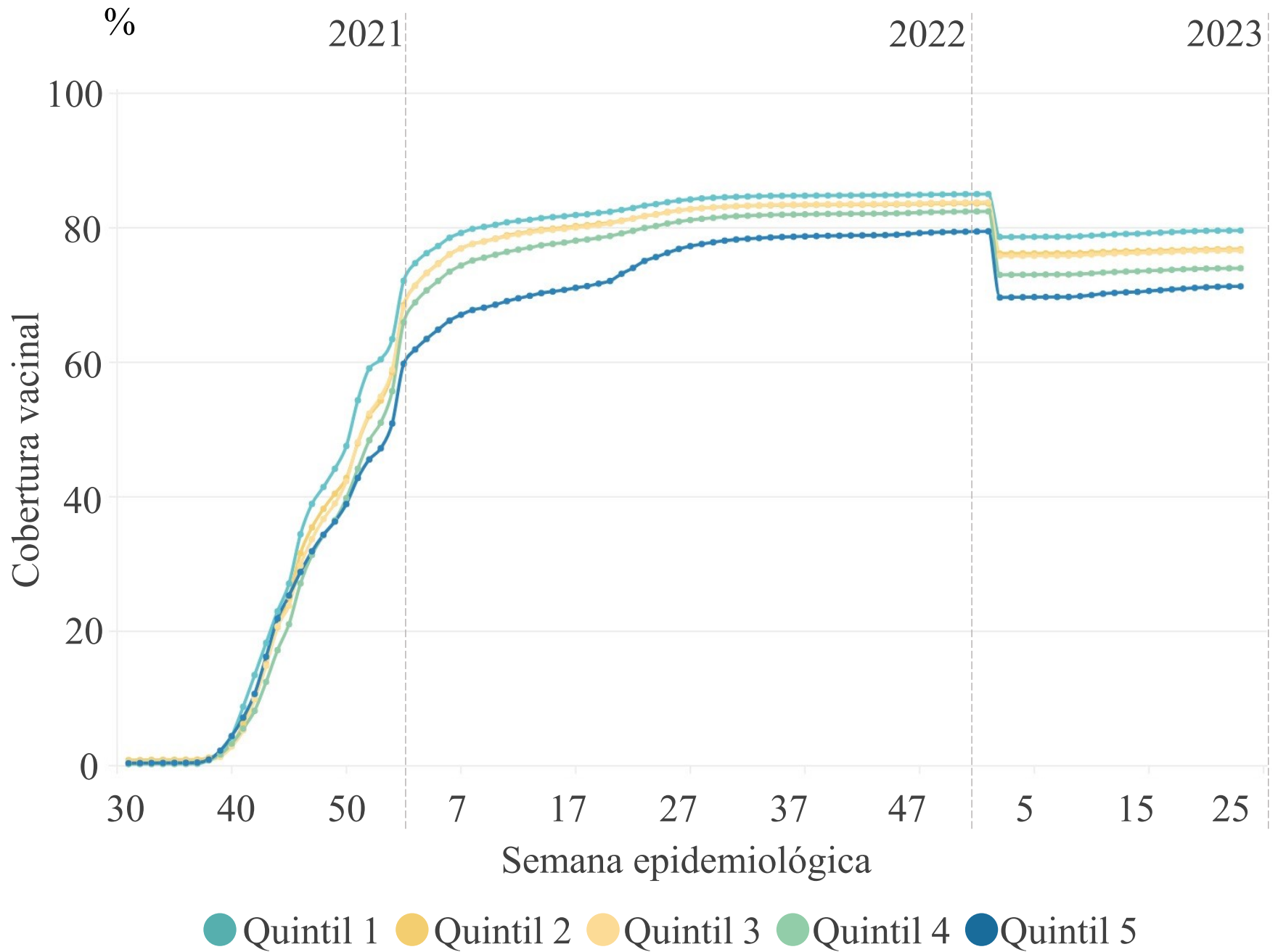

# C1

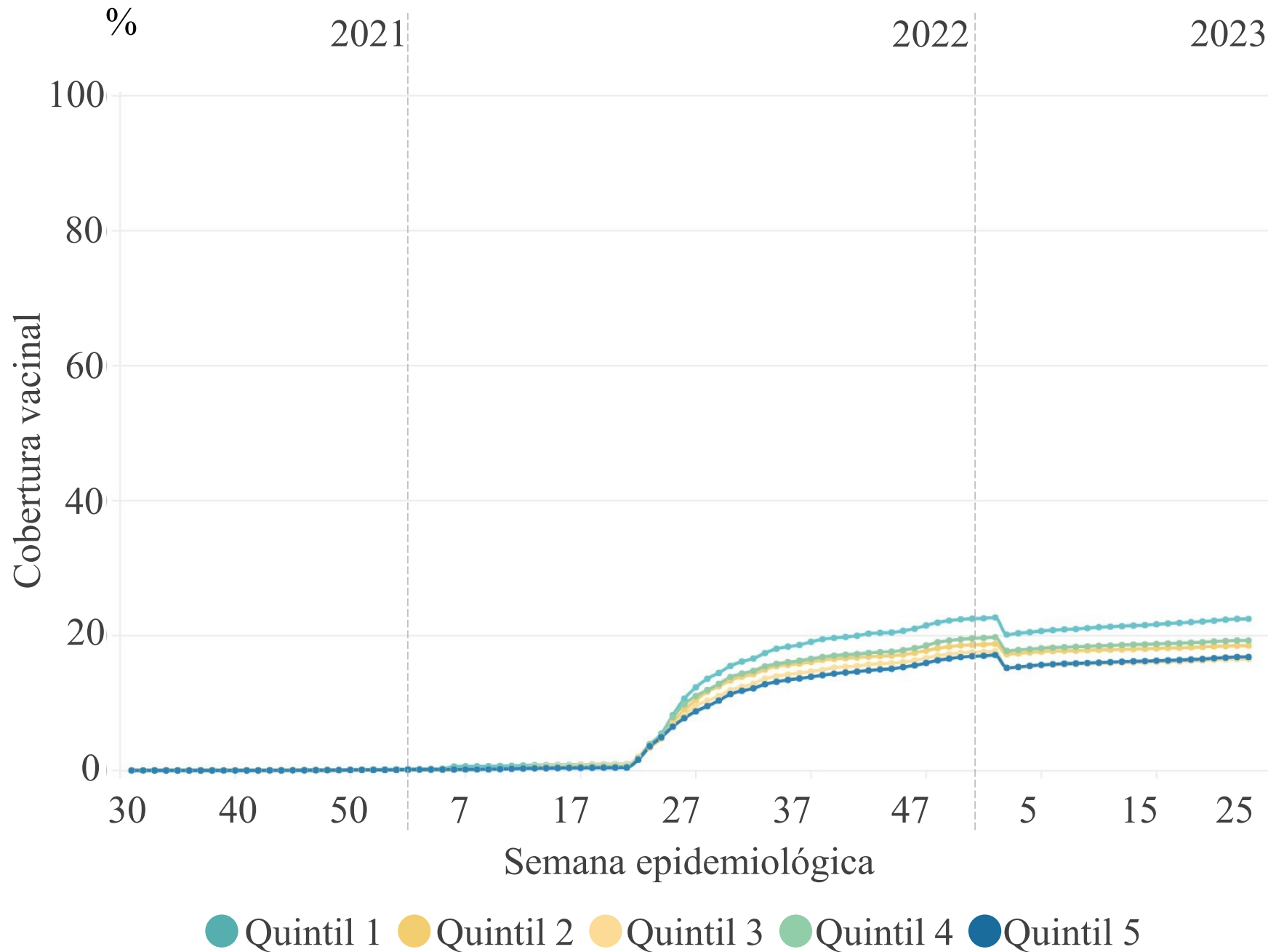

# C2

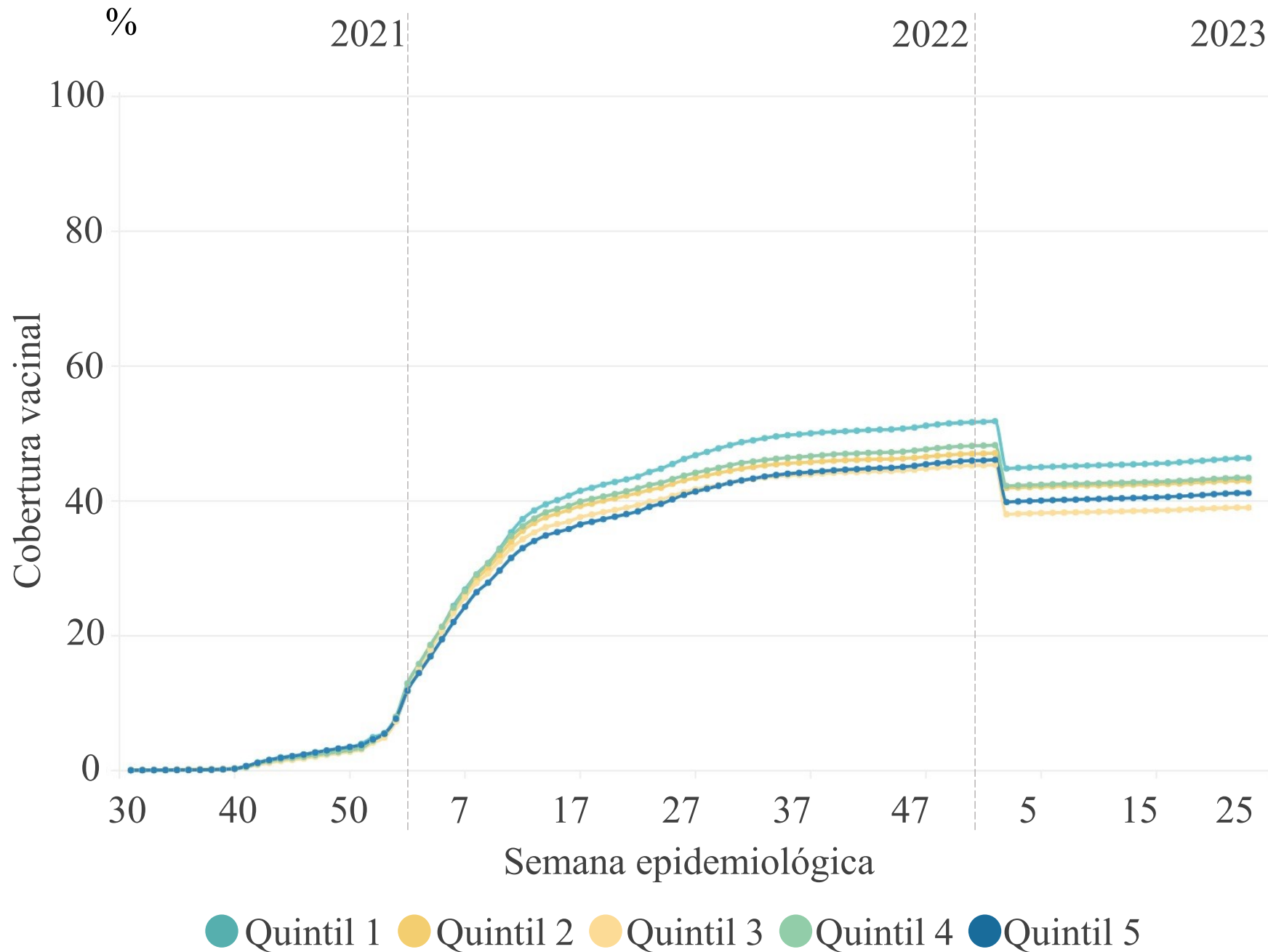

# C3

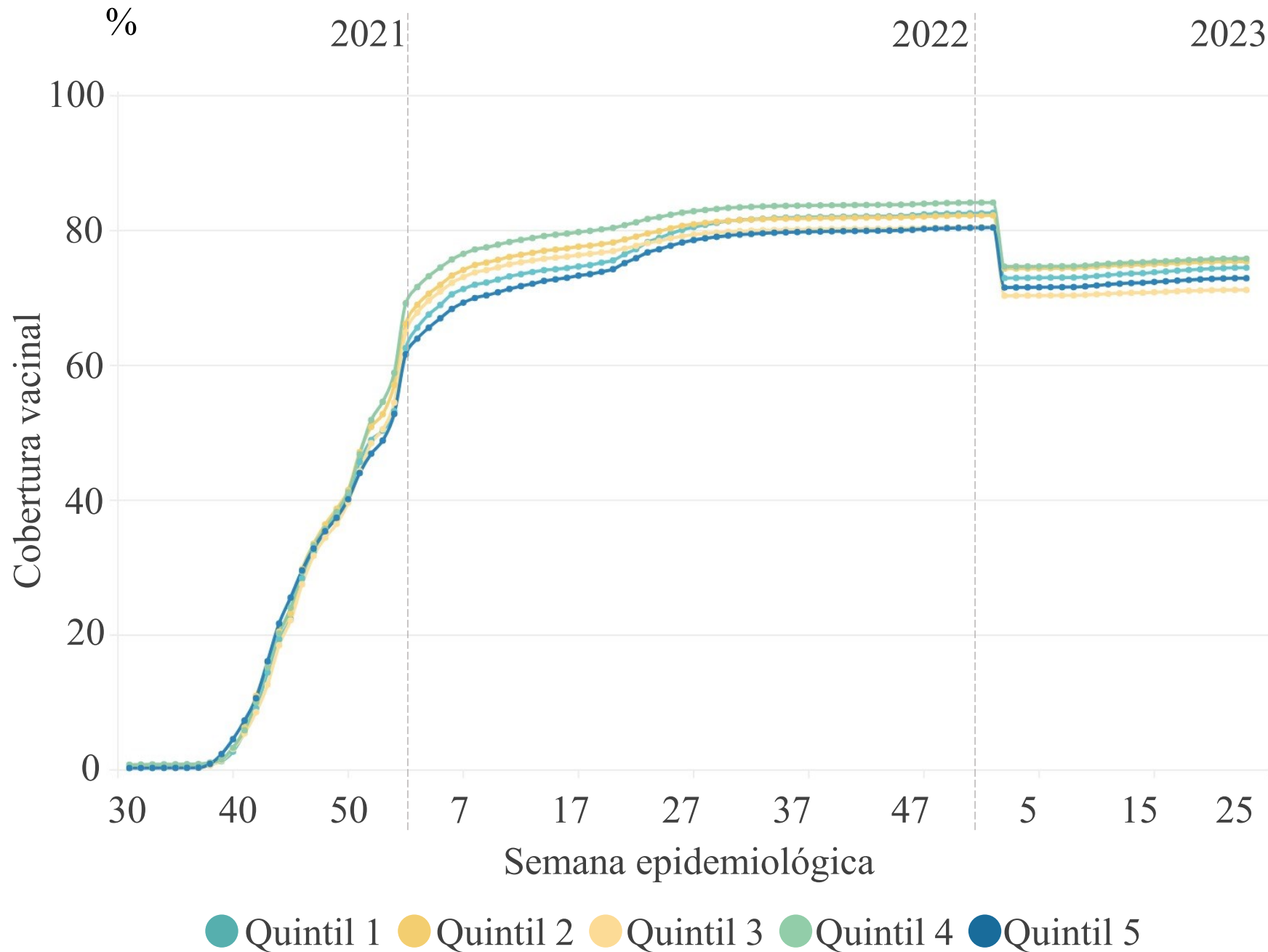

Supplement: Supplementary file 5 [file 2237-9622-ress-34-e20240329-supp03-pt.pdf]
