# Supplementary material for: Intrastate inequality of COVID-19 vaccination coverage: spatial analysis and socioeconomic, Santa Catarina, 2021-2023
Source: Epidemiol Serv Saude. 2025 Aug 8;34:e20240329. doi: 10.1590/S2237-96222025v34e20240329.en (PMC12342717; doi:10.1590/S2237-96222025v34e20240329.en)
Supplement: Translation

# Translation: Desigualdade intraestadual da cobertura vacinal contra a covid-19: análise espacial e segundo condições socioeconômicas, Santa Catarina, 2021-2023

**DOI:** 10.1590/S2237-96222025v34e20240329.pt

**Authors:** Clorine Borba Zanlourensi, Antônio Fernando Boing

**Affiliations:** 1 Universidade Federal de Santa Catarina, Centro de Ciências da Saúde, Programa de Pós-Graduação em Saúde Coletiva, Florianópolis, SC, Brasil

**Keywords:** Vacinação, Covid-19, Indicadores de Desigualdade em Saúde, Fatores Sociodemográficos, Saúde Pública

## Abstract

**Objetivo:**

Analisar a distribuição da cobertura vacinal contra a covid-19 em Santa Catarina e sua associação com aspectos socioeconômicos dos municípios entre 2021 e 2023.

**Métodos:**

Foram analisados dados de indivíduos a partir de 12 anos, com esquema vacinal completo com três doses. As variáveis independentes incluíram dados educacionais, de desenvolvimento humano municipal e composição racial dos municípios. Analisaram-se a evolução e a distribuição espacial da cobertura vacinal, identificando agrupamento de municípios e calculando razão e diferença absolutas entre maiores e menores quintis de cobertura vacinal.

**Resultados:**

A cobertura vacinal no estado foi 26,9% para jovens, 50,3% para adultos e 78,8% para idosos. Municípios com maior cobertura vacinal apresentaram 5,4 vezes mais população vacinada entre 12 e 17 anos, 2,1 vezes mais na faixa etária de 18-59 anos e 1,4 vez mais na população com 60 anos ou mais. Municípios com maior nível educacional, menor proporção de população negra e menor índice de desenvolvimento humano municipal apresentaram maior cobertura vacinal. Essas disparidades foram persistentes. Aglomerados de municípios com alta vacinação foram encontrados.

**Conclusão:**

Houve disparidades na cobertura vacinal do estado, com associação de aspectos socioeconômicos dos municípios.

**Aspectos éticos**

Esta pesquisa utiliza dados abertos. Segundo a Resolução n° 510/2016 do Conselho Nacional de Saúde, pesquisas que utilizam dados de acesso público não precisam da apreciação por parte do comitê de ética, pois esses dados já estão anonimizados e acessíveis ao público.:

## Introdução

A vacinação contra a covid-19 é considerada uma medida de saúde pública crucial para reduzir as taxas de hospitalização e mortalidade pela doença (1). As vacinas mostraram alta eficácia na prevenção de formas graves da covid-19. Isso evidencia que a vacinação em larga escala desempenhou papel fundamental no controle da pandemia dessa enfermidade e na proteção individual (2). Além do esquema vacinal primário, com duas doses, doses de reforço foram recomendadas para prevenir covid-19 grave e fatal ainda em 2021 para todos os grupos etários (3). 

Até maio de 2024, estimou-se que 67,0% da população mundial completou a série primária e 32,0% recebeu pelo menos uma dose de reforço da vacina contra a covid-19 (4). Além de baixa, essa cobertura vacinal foi considerada desigual, com o percentual de cobertura de duas doses chegando a 79,86% entre residentes de países de alta renda e a 32,82% da população nos países de baixa renda (5).

No Brasil, de 2020 a 2022, houve marcante descoordenação nacional nas ações de enfrentamento à covid-19, e muitas condutas não foram orientadas pelas melhores evidências científicas. Mesmo que o país contasse com o Programa Nacional de Imunização, reconhecido internacionalmente como um dos mais completos e capilarizados do mundo, houve atrasos na aquisição de vacinas contra a covid-19 e forte campanha de descredibilização da vacinação (6). Até maio de 2024, verificou-se que 84,6% dos brasileiros completaram o esquema vacinal primário, enquanto 52,8% receberam uma dose de reforço, havendo forte variação de acordo com a faixa etária da população (7). Apesar do princípio de equidade do Sistema Único de Saúde, o acesso aos serviços de saúde no Brasil apresentou desigualdades regionais, socioeconômicas e demográficas (8). 

A escassa literatura que investigou as desigualdades na vacinação contra covid-19 no Brasil esteve concentrada em análises de todo o território nacional, havendo poucos estudos com análises espaciais intraurbanas e intraestaduais (8-10). Indicadores que estimam médias de agregados geográficos maiores podem ocultar importantes disparidades em seu interior. Análises com dados mais agregados e sem recortes regionais podem ocultar desigualdades significativas, como entre diferentes municípios ou regiões dentro dos estados. 

Conhecer e compreender as disparidades regionais é fundamental para desenhar políticas públicas mais eficazes e promover oferta mais equitativa de insumos e ações em saúde pública. Em junho de 2024, Santa Catarina ocupava a 17ª posição em cobertura vacinal entre as unidades federativas do Brasil. Não foram encontrados estudos que avaliavam, de forma sistematizada, sua distribuição intraestadual (11). Tal lacuna inviabiliza a análise do papel governamental no cumprimento do princípio de equidade do sistema de saúde, em especial em momento de emergência sanitária.

O objetivo deste estudo foi analisar a distribuição da cobertura vacinal contra a covid-19 em Santa Catarina e sua associação com aspectos socioeconômicos dos municípios entre 2021 e 2023.

## Métodos

### Delineamento do estudo e contexto

Tratou-se de estudo observacional, ecológico e longitudinal. Os dados obtidos são referentes à vacinação contra a covid-19 nos municípios de Santa Catarina entre 16 de janeiro de 2021 e 3 de junho de 2023.

### Participantes

Todos os indivíduos a partir de 12 anos de idade residentes em Santa Catarina e vacinados entre 16 de janeiro de 2021 e 3 de junho de 2023 foram incluídos no estudo. Os registros foram classificados em três grupos etários: adolescentes (12-17 anos), adultos (18-59 anos) e idosos (60 anos ou mais).

### Variáveis

A variável dependente foi a vacinação completa com três doses: primeira dose, segunda dose e dose de reforço. Os registros foram classificados segundo a semana epidemiológica, a ordem de aplicação das vacinas e o município de residência.

As variáveis independentes foram os indicadores sociodemográficos. O indicador educacional utilizado foi a quantidade de anos de estudo esperados até os 18 anos de idade entre os residentes de cada município, sob a premissa de que os padrões de escolarização observados quando da coleta de dados se mantenham ao longo de suas vidas. A composição racial de cada município foi determinada pela proporção de residentes pretos em relação ao total de residentes. O índice de desenvolvimento humano municipal (IDHM) é uma medida que avalia o desenvolvimento humano em cada município, considerando três indicadores principais: educação, longevidade e renda. Essas variáveis foram divididas em quintis com base na distribuição entre os municípios.

### Fontes de dados

Foram analisados os registros de vacinação do Sistema de Informações do Programa Nacional de Imunizações (SI-PNI). No Brasil, todas as doses de vacinas aplicadas contra a covid-19 são compulsoriamente registradas pelos serviços de saúde para envio ao Ministério da Saúde. Este consolida os dados de todo o território nacional e os disponibiliza publicamente na plataforma openDataSUS. Todos os bancos de dados apresentam informações anonimizadas a respeito da administração de doses das vacinas. Esse conjunto de dados inclui informações sobre o município e o estado, a data da aplicação, o tipo de vacina administrada, o sexo e a idade das pessoas vacinadas. Os dados de vacinação podem ser acessados no seguinte repositório: https://github.com/covid19br/dados-vacinas. 

Para calcular a cobertura vacinal, utilizou-se como denominador o conjunto de dados sobre população residente, conforme disponibilizado pelo Instituto Brasileiro de Geografia e Estatística, referentes aos Censos Demográficos 2010 (https://sidra.ibge.gov.br/tabela/1378) e 2022 (https://sidra.ibge.gov.br/tabela/9514).

Os indicadores sociodemográficos foram obtidos pelo Censo Demográfico 2010, ano mais recente com dados socioeconômicos municipais consolidados e disponíveis no Brasil em junho de 2024. Esses indicadores foram divulgados no Atlas do Desenvolvimento Humano do Brasil (https://www.atlasbrasil.org.br/). 

### Viés

Por utilizar dados secundários, este estudo pode ter inconsistências e erros advindos de falhas no registro de informações da vacinação no SI-PNI. O uso do Censo Demográfico 2010, o mais recente disponível no momento do levantamento de dados, pode apresentar imprecisões devido a mudanças e alterações demográficas com o passar do tempo.

### Tamanho do estudo

O estudo incluiu todos os registros de vacinação contra a covid-19 de indivíduos acima de 12 anos, que estavam disponíveis no SI-PNI, de 16 de janeiro de 2021 a 3 de junho de 2023. Dados de indivíduos de 0-11 anos foram excluídos do banco de dados.

### Métodos estatísticos

Para calcular a cobertura vacinal, dividiu-se o número de vacinados pela população residente em cada faixa etária dentro de cada município, e o resultado foi multiplicado por 100. Foram estimados os valores de três doses completas em todas as semanas epidemiológicas de 2021 até a 22ª semana epidemiológica de 2023 (16 de janeiro de 2021 a 3 de junho de 2023). 

Mapas da distribuição espacial da cobertura vacinal, segundo os municípios de Santa Catarina, foram produzidos utilizando-se o *software* QGIS 3.34.0 (Quantum Geographic Information System). 

Os municípios foram divididos em quintis de acordo com as variáveis sociodemográficas, e calcularam-se as coberturas vacinais em cada faixa etária. Cada quintil representa 20,0% da distribuição de cobertura vacinal, do menor para o maior. Também foram calculadas a razão do desfecho segundo os quintis – dividindo-se o valor de cobertura vacinal do maior quintil pelo valor do menor quintil – e a diferença absoluta – subtraindo-se a cobertura vacinal do quintil de maior valor pelo observado no quintil de menor valor. 

Para investigar a existência de aglomerados espaciais, foi calculado o índice de Moran local univariado (Local Indicators of Spatial Association – LISA). Esse método analisou a autocorrelação espacial dos dados, identificando regiões nas quais os valores semelhantes estão mais próximos entre si do que seria esperado ao acaso. 

Foram identificadas quatro classes de aglomerados estatisticamente significativas: i) alto-alto, em que há intensidade de cobertura vacinal entre uma área e seus vizinhos; ii) baixo-baixo, em que há baixa cobertura vacinal entre uma área e seus vizinhos; iii) baixo-alto, a qual reflete áreas com valores baixos de cobertura vacinal cercadas por áreas com valores altos; e iv) alto-baixo, que indica um ponto isolado de alta cobertura vacinal, destoando do padrão circundante. A análise espacial e a confecção dos mapas foram realizadas no software GeoDa 1.22.0.4. As análises estatísticas foram realizadas no Stata 15.1.

## Resultados

Até a semana epidemiológica 22 de 2023, foram administradas 14.872.425 doses da vacina contra a covid-19 em residentes de Santa Catarina. Os idosos foram o grupo com maior cobertura vacinal (CV) das três doses (78,8%, CV 10,7%), seguidos pelos adultos (50,3%, CV 15,1%) e pelos adolescentes (26,9%, CV 18,6%).

A cobertura vacinal com três doses variou substancialmente entre os municípios. A macrorregião do oeste catarinense se destacou, especialmente a microrregião de São Miguel do Oeste, com os maiores índices de cobertura nos municípios nas três categorias de idade. As macrorregiões do norte catarinense e do Vale do Itajaí registraram os menores índices de cobertura (Figura 1). Observam-se as divisões geográficas de cada macrorregião do estado (Figura Suplementar 1) e apresentam-se as coberturas vacinais ao longo do período de análise, notando-se que as diferenças regionais se estabeleceram desde o início da campanha de vacinação até o final do período analisado (Figura Suplementar 2).

**Figure 1:** Evolution of COVID-19 vaccination coverage, by municipality vaccination coverage^a^. Santa Catarina, 2021-2023 (n=295)

(A) população de 12-17 anos de idade; (B) população de 18-59 anos de idade; (C) população com 60 anos de idade ou mais; aSemana epidemiológica 22 de 2023 é referente a 3 de junho de 2023. Santa Catarina, 2023, 295 municípios.

Foi verificada profunda disparidade na cobertura vacinal entre os municípios quando agrupados em quintis de cobertura (Tabela 1). O quintil 1, com a menor cobertura vacinal, registrou 10,8% de população com as três doses para a faixa etária de 12-17 anos, 36,2% para a faixa etária de 18-59 anos e 73,1% para indivíduos com 60 anos ou mais. O quintil 5 compreendeu os municípios com a maior cobertura vacinal. Este apresentou valores iguais a 58,2% entre aqueles com 12-17 anos, 77,5% para a população de 18-59 anos e 104,2% para 60 anos ou mais. Os municípios do quintil com maior expectativa de escolaridade aos 18 anos apresentaram a maior cobertura vacinal em todas as faixas etárias. Maior cobertura vacinal também foi observada nos quintis de municípios com menor percentual de população negra. O padrão foi distinto no que se refere ao IDHM; o quintil 5, com o maior IDHM, teve a maior cobertura vacinal.

**Tabela 1:** Proporção da cobertura vacinal contra a covid-19 segundo os quintis entre os municípios, e os indicadores socioeconômicos. Santa Catarina, Semana Epidemiológica 22 de 2023^a^ (n=295)

Variável socioeconômica	12-17 anos n (%)	18-59 anos n (%)	60+ anos n (%)
**Municípios**			
Quintil^b^ 1 (menor)	11.132 (10,8)	612.836 (36,2)	272.298 (73,1)
Quintil^b^ 2	29.811 (16,0)	429.590 (46,1)	262.103 (81,5)
Quintil^b^ 3	31.643 (23,2)	585.289 (54,9)	211.169 (88,2)
Quintil^b^ 4	15.819 (32,9)	262.442 (63,5)	88.222 (94,6)
Quintil^b^ 5 (maior)	10.928 (58,2)	113.840 (77,5)	50.108 (104,2)
**Anos esperados de escolaridade aos 18 anos**			
Quintil^b^ 1 (menor)	11.366 (20,8)	191.315 (44,8)	87.175 (82,0)
Quintil^b^ 2	26.943 (17,5)	553.324 (42,9)	244.354 (79,1)
Quintil^b^ 3	27.930 (18,5)	610.320 (47,6)	270.421 (83,2)
Quintil^b^ 4	23.868 (24,6)	500.708 (57,2)	206.580 (85,0)
Quintil^b^ 5 (maior)	8.890 (29,5)	142.183 (58,4)	71.635 (91,2)
**Proporção de população preta**			
Quintil^b^ 1 (menor)	6.868 (28,8)	102.505 (54,2)	54.668 (86,1)
Quintil^b^ 2	9.741 (22,2)	173.850 (51,1)	84.807 (84,4)
Quintil^b^ 3	14.595 (22,2)	272.878 (51,1)	125.656 (84,7)
Quintil^b^ 4	20.376 (19,8)	401.075 (46,7)	183.416 (83,6)
Quintil^b^ 5 (maior)	47.753 (18,9)	1.053.689 (47,8)	435.353 (81,4)
**Índice de desenvolvimento humano municipal** (IDHM)			
Quintil^b^ 1 (menor)	12.199 (25,5)	208.761 (53,7)	87.150 (84,4)
Quintil^b^ 2	10.737 (20,3)	200.921 (48,3)	97.046 (83,4)
Quintil^b^ 3	12.475 (19,4)	240.075 (46,6)	110.466 (81,6)
Quintil^b^ 4	16.212 (21,7)	302.754 (49,7)	137.219 (85,5)
Quintil^b^ 5 (maior)	47.294 (19,2)	1.043.603 (47,8)	447.164 (82,0)

^a^Semana epidemiológica 22 de 2023 é referente a 3 de junho de 2023; ^b^Quintil: dados divididos em cinco partes que representam 20,0% da distribuição de cobertura vacinal, do menor para o maior (do 1º ao 5º quintil). Santa Catarina, 2023, 295 municípios.

As desigualdades absolutas e a razão entre os quintis extremos dos indicadores sociodemográficos, ao longo da campanha de vacinação, revelaram disparidades significativas, especialmente entre os quintis extremos (Tabela 2). Os municípios com maior cobertura vacinal demonstraram ter 5,4 vezes mais população vacinada entre aqueles com idade entre 12 e 17 anos, 2,1 vezes mais na faixa etária de 18-59 anos e 1,4 vez mais na população com 60 anos ou mais. O indicador socioeconômico de anos de estudo apresentou maior desigualdade na cobertura vacinal em todas as categorias de idade, especialmente entre os jovens de 12-17 anos (Tabela 2).

**Tabela 2:** Razão, diferenças relativas e absolutas de cobertura vacinal contra a covid-19 por extrato de idade, comparando os valores dos quintis extremos (quintil 5 e quintil 1) entre a cobertura vacinal nos municípios e os indicadores socioeconômicos. Santa Catarina, Semana Epidemiológica 22 de 2023^a^ (n=295)

Indicadores socioeconômicos (anos)	Razão da cobertura vacinal (Q5^b^/Q1^c^)	Diferença absoluta da cobertura vacinal (%) ( Q5^b^/Q1^c^)
Municípios		
12-17	5,39	47,4
18-59	2,14	41,3
60+	1,43	31,1
**Tempo de escolaridade aos 18 anos**		
12-17	1,42	8,7
18-59	1,30	13,6
60+	1,11	9,3
**Proporção de população preta**		
12-17	0,66	-9,9
18-59	0,88	-6,5
60+	0,95	-4,7
**Índice de desenvolvimento humano municipal** (IDHM)		
12-17	0,75	-6,4
18-59	0,89	-6,0
60+	0,97	-2,3

^a^Semana epidemiológica 22 de 2023 é referente a 3 de junho de 2023; ^b^Q5: quintil 5; ^c^Q1: quintil 1. Santa Catarina, 2023, 295 municípios.

Durante a campanha de vacinação, os quintis de municípios que registraram as mais altas coberturas vacinais no início mantiveram-se à frente com coberturas maiores do que aqueles com início de cobertura mais baixa, independentemente da faixa etária (Figura 2). O mesmo padrão foi observado quando analisadas as coberturas segundo as variáveis sociodemográficas (Figura Suplementar 3).

**Figure 2:** Evolução da cobertura vacinal contra covid-19, segundo quintila de cobertura vacinal dos municípios. Santa Catarina, 2021-2023 (n=295)

(A) população de 12-17 anos de idade; (B) população de 18-59 anos de idade; (C) população com 60 anos de idade ou mais; aQuintil: dados divididos em cinco partes que representam 20,0% da distribuição, do menor para o maior (do 1º ao 5º quintil). Santa Catarina, 2023, 295 municípios.

Na autocorrelação espacial, foram observados aglomerados alto-alto estatisticamente significativos, o que indicou altas taxas de cobertura vacinal em áreas onde os vizinhos também apresentam altos valores (Figura 3). Esses aglomerados estavam localizados principalmente na macrorregião oeste catarinense, abrangendo todas as faixas etárias. Os aglomerados baixo-baixo, que indicam baixa cobertura vacinal com vizinhos em condições semelhantes, concentraram-se principalmente no norte catarinense e no Vale do Itajaí.

**Figure 3:** Autocorrelação espacial da cobertura vacinal contra covid-19, de acordo com o índice de Moran local univariado. Santa Catarina, Semana Epidemiológica 22de 2023a (n=295)

(A) população de 12-17 anos de idade; (B) população de 18-59 anos de idade; (C) população com 60 anos de idade ou mais. aSemana epidemiológica 22 de 2023 é referente a 3 de junho de 2023. Santa Catarina, 2023, 295 municípios.

## Discussão

Este estudo identificou expressivas disparidades na cobertura vacinal contra covid-19 entre os municípios de Santa Catarina. Os municípios com maior nível educacional, menor proporção de população negra e menor IDHM apresentaram maior cobertura vacinal contra a covid-19. Ao comparar os municípios, a vacinação foi mais desigual entre os jovens e menor entre os idosos. Essas disparidades se mantiveram persistentes desde o início até o final do período investigado. Foram encontrados aglomerados de municípios com altas taxas de vacinação na região oeste catarinense e com baixa vacinação nas regiões norte catarinense e Vale do Itajaí.

Houve discrepância da cobertura vacinal entre diferentes regiões e municípios em nível nacional (8,12).

Os municípios brasileiros apresentaram o nível de agregação que concentrou a maior parte da variabilidade do desfecho em comparação a estados e regiões, principalmente na terceira dose da vacina (8). Neste estudo, desigualdades entre os municípios foram consistentes em todas as faixas etárias avaliadas e persistiram ao longo de todas as semanas epidemiológicas. Também foram encontrados aglomerados de municípios com maiores e menores coberturas vacinais. Isso pode indicar que alguns municípios compartilham características espaciais semelhantes as quais favoreceram a alta cobertura, enquanto outros enfrentam desafios comuns que prejudicaram a vacinação.

Esses aspectos podem estar relacionados à infraestrutura de saúde que pode ter dificultado a logística da campanha de vacinação e o acesso aos serviços em algumas localidades (13). Instalações de saúde inadequadas podem estar associadas à falta de recursos financeiro suficientes para investir em melhores estruturas, equipamentos e treinamento de pessoal (14). Regiões brasileiras com piores indicadores de renda podem encontrar dificuldades em garantir recursos adequados para infraestrutura de saúde, o que pode prejudicar a administração eficaz e acessível de vacinas à população (12). 

Existem outros obstáculos, como o acesso aos serviços de saúde e a resistência à vacinação entre a população local (13,15). Moradores de municípios brasileiros com renda mais baixa podem ter enfrentado maiores desafios financeiros ao tentar acessar os locais de vacinação. Também pode haver níveis mais baixos de confiança nas autoridades de saúde e, consequentemente, nas recomendações de vacinação (12). Um levantamento realizado em 14 países demonstrou que o acesso a serviços de saúde preventivos está positivamente associado a uma maior taxa de vacinação contra a covid-19 (16). A vacinação pode ser influenciada pela qualidade das experiências recentes dos usuários de serviços de cuidados de saúde (16). 

Os profissionais de saúde desempenham papel crucial na disseminação de informações confiáveis sobre a vacinação (16,17). A confiança dos pacientes no cuidado recebido fortalece o vínculo com a equipe de saúde, incentivando-os a procurar os serviços de saúde e a participar das campanhas de vacinação (13). A falta de recursos humanos suficientes e de oportunidades de formação dos profissionais de saúde pode ocasionar dificuldades na administração das doses, assim como na manutenção dos registros no SI-PNI (15). 

A operacionalização das campanhas de vacinação no Brasil ocorre em nível municipal, o que, primariamente, torna responsabilidade das prefeituras identificar e superar obstáculos na imunização da população. Embora essa responsabilidade local seja importante, a participação ativa do governo estadual assume papel fundamental no sucesso das campanhas. Além de aprimorar a distribuição das vacinas e identificar municípios com cobertura vacinal deficiente, o governo estadual deve oferecer apoios técnico e financeiro necessários (8). Vale ressaltar a lacuna de ações durante os anos de emergência sanitária para mitigar as desigualdades na cobertura vacinal, que foram persistentes ao longo do tempo. Para enfrentar essas disparidades, é essencial que o governo estadual se engaje ativamente no processo de vacinação, coordenando esforços e alocando recursos de forma eficiente para garantir a cobertura vacinal abrangente e equitativa em todo o estado. Essa atuação é primordial para identificar agrupamentos regionais que apresentam desafios e demandam abordagem diferenciada. 

No contexto das desigualdades socioeconômicas, diferentes países apresentaram associação da maior taxa de vacinação contra a covid-19 com o maior nível de escolaridade (18,19). No Brasil, até 2022 a cobertura populacional de doses de reforço era mais elevada em municípios com maior expectativa de escolaridade aos 18 anos (58,7%) em comparação com aqueles de menor nível educacional (41,2%) (8). Os achados deste estudo corroboram as pesquisas apresentadas até o momento, pois, em todas as faixas etárias, foram encontradas disparidades entre os municípios segundo o nível educacional. A relação entre a educação populacional e a cobertura vacinal pode ser explicada pela maior capacidade das pessoas com maior nível de escolaridade em acessar informações sobre saúde e confiar mais na ciência e nas recomendações de saúde pública, o que pode aumentar sua disposição para se vacinar (20,21). 

Os municípios com maior proporção populacional com níveis mais elevados de escolaridade podem apresentar maior cobertura vacinal. Adolescentes e adultos brasileiros com menor nível educacional têm maior hesitação à vacinação (22). Municípios com população mais escolarizada também podem ser aqueles com melhor estrutura prévia dos serviços de saúde, menos barreiras de acesso, mais legitimidade dos profissionais de saúde, os quais ofereçam condições objetivas e materiais mais propícios à vacinação. Uma estratégia para contemplar a disseminação do conhecimento com base científica sobre as vacinas de covid-19, para toda a população, é a divulgação de informações em mídias tradicionais e eletrônicas e a produção de materiais de apoio, como vídeos e cartilhas (23).

O estudo revelou que municípios com maior proporção de população preta apresentaram menor cobertura vacinal. No Brasil, os municípios com maior proporção de população negra tiveram maior probabilidade de ter menor cobertura vacinal (8,12). Em Michigan, nos Estados Unidos, bairros com maior proporção de população negra também apresentaram menores taxas de vacinação contra a covid-19 (24). Locais com maior proporção de população negra podem apresentar níveis mais altos de pobreza, desemprego, baixa escolaridade e menor acesso a serviços de saúde, incluindo a disponibilidade de locais de vacinação e transporte. Todos esses fatores podem afetar o acesso e a procura pela vacinação (25). O engajamento das três esferas de gestão do Sistema Único de Saúde na ampliação de salas de vacina, a busca ativa e a mobilização de profissionais de saúde em áreas que apresentam maior desigualdade social podem ser estratégias eficazes para o acesso à vacinação de forma equitativa (23). 

A cobertura vacinal dos municípios de maior IDHM foi menor quando comparada aos de maior IDHM. Na Paraíba, o maior percentual de indivíduos hospitalizados com esquema vacinal completo ocorreu em municípios com IDHM baixo e médio (26). Uma análise dos 5.570 municípios brasileiros mostrou que a cobertura vacinal era menor em locais com baixo IDHM. Nos municípios com rede sólida de atenção primária à saúde, a cobertura vacinal pode se aproximar dos níveis observados em áreas com alto IDHM, destacando a importância da atenção primária para melhorar a vacinação em regiões menos desenvolvidas (27). 

O IDHM pode esconder disparidades regionais internas, nas quais algumas áreas enfrentam altos níveis de desigualdade e acesso desigual aos serviços de saúde. Municípios com IDHM mais baixo, mas com campanhas de vacinação bem direcionadas, podem alcançar taxas de cobertura mais altas (28). O IDHM é a composição de indicadores de longevidade, educação e renda. Porém, é possível que um município tenha um alto IDHM devido à longevidade e renda, mas enfrente desafios significativos em termos de educação, e vice-versa. Essa complexidade pode ajudar a explicar os resultados do estudo (28).

Este estudo apresentou algumas limitações. Os dados de composição racial e socioeconômicos foram provenientes do Censo Demográfico 2010, o último ano disponível no momento da análise. Municípios podem ter melhorado ou piorado seus indicadores comparativamente ao longo de uma década e terem sido posicionados em um quintil diferente do cenário atual. O registro das doses da vacina pelos municípios enfrentou problemas durante a pandemia de covid-19, como atrasos e erros no preenchimento devido à falta de treinamento e monitoramento adequado para os profissionais de saúde sobre a importância e a operacionalização dos dados, problemas nos sistemas locais e na centralização dos dados e acesso limitado à internet em áreas remotas. Foram utilizados os dados oficiais de vacinação do Brasil e, ainda que haja necessidade de aperfeiçoamento, os dados do SI-PNI são os mais completos e confiáveis disponíveis, sendo monitorados continuamente para identificar e corrigir erros. As informações são disponibilizadas de forma transparente e permitem monitoramento abrangente das campanhas de vacinação.

A análise das disparidades na cobertura vacinal contra a covid-19 em Santa Catarina revelou padrões preocupantes. Essas disparidades persistiram ao longo do tempo, indicando que são questões estruturais que requerem intervenções contínuas e políticas públicas direcionadas. A identificação de aglomerados com altas taxas de vacinação na região oeste, em contraste com baixas taxas no norte e no Vale do Itajaí, sugere a necessidade de investigar e abordar as causas específicas dessas disparidades regionais. Para mitigar essas desigualdades, é importante que haja monitoramento da cobertura vacinal segundo as dimensões sociais, econômicas e espaciais. 

É importante que a informação gerada pelo monitoramento subsidie apoios técnicos e financeiros aos municípios, reforço em sua infraestrutura e de profissionais atuantes no Sistema Único de Saúde e ampliação do acesso aos serviços de saúde. Ações de planejamento local focados nesses municípios de menor cobertura devem ser conduzidas para indicar as ações específicas mais necessárias ao contexto. Esses achados do estudo reforçam a importância da participação ativa do estado e a implementação de políticas públicas que visem reduzir as desigualdades socioeconômicas e regionais na cobertura vacinal, garantindo o acesso equitativo à vacinação contra a covid-19 em toda a população de Santa Catarina.

## Data Availability

O banco de dados de vacinação pode ser acessado no seguinte repositório: https://github.com/covid19br/dados-vacinas. Os dados populacionais referentes ao Censo Demográfico 2010 podem ser acessado em: https://sidra.ibge.gov.br/tabela/1378 e ao Censo Demográfico 2022 em: https://sidra.ibge.gov.br/tabela/9514.

## Material Suplementar

Figura Suplementar 1

Figura Suplementar 2

Figura Suplementar 3
